# Supplementary material for: Actin Depletion Initiates Events Leading to Granule Secretion at the Immunological Synapse
Source: Immunity. 2015 May 19;42(5):864–76. doi: 10.1016/j.immuni.2015.04.013 (PMC4448150; doi:10.1016/j.immuni.2015.04.013)
Supplement: Document S1. Figures S1–S3 [file mmc1.pdf]

Immunity

Supplemental Information

Actin Depletion Initiates Events Leading to Granule Secretion at the Immunological Synapse

Alex T. Ritter, Yukako Asano, Jane C. Stinchcombe, N.M.G. Dieckmann, Bi-Chang Chen, C. Gawden-Bone, Schuyler van Engelenburg, Wesley Legant, Liang Gao, Michael W. Davidson, Eric Betzig, Jennifer Lippincott-Schwartz, and Gillian M. Griffiths

Figure S1

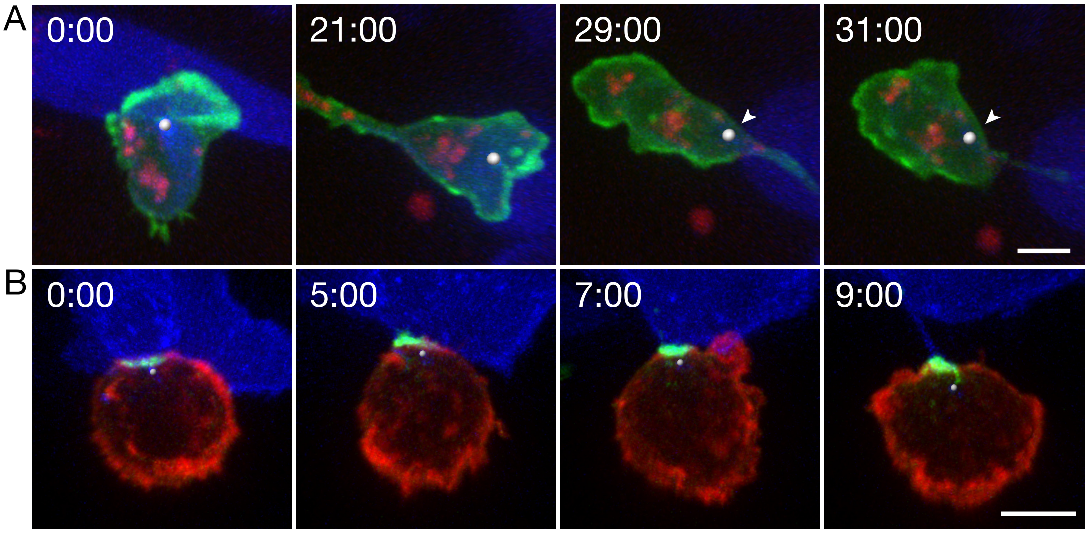

Figure S1: CTL assume the morphology of a migrating cell when retracting from target, related to Figure 6. (A) Timelapse of maximum intensity projection images of a CTL expressing Lifeact-EGFP, CD63-mCherry, and MAPTau-tagBFP2 interacting with a MC57 target cell (blue), (n=13). The location of the centrosome is marked with a white sphere. Arrows highlight the location of the centrosome in the uropod upon retraction. (B) Timelapse of maximum intensity projection images of a CTL expressing CD3ζ-EGFP, Lifeact-mApple and PACT-tagBFP (marked with a white sphere) interacting with an EL4 target cell (blue). Scale bar: 5µm. Time in min**.**

Figure S2

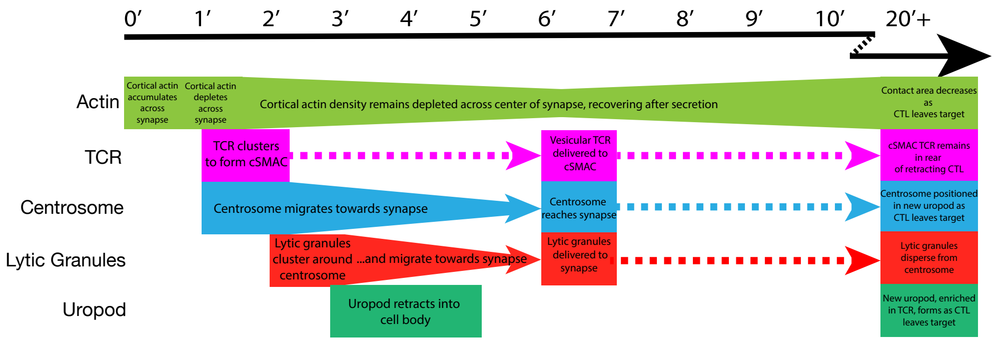

Figure S2, related to Discussion: The order of events leading to secretion at the immunological synapse. Timeline (in min) showing the order of events after a CTL encounters a cognate target with the reorganization of cortical actin, TCR, centrosome, granules and uropod depicted relative to one another as described in the text.

Figure S3

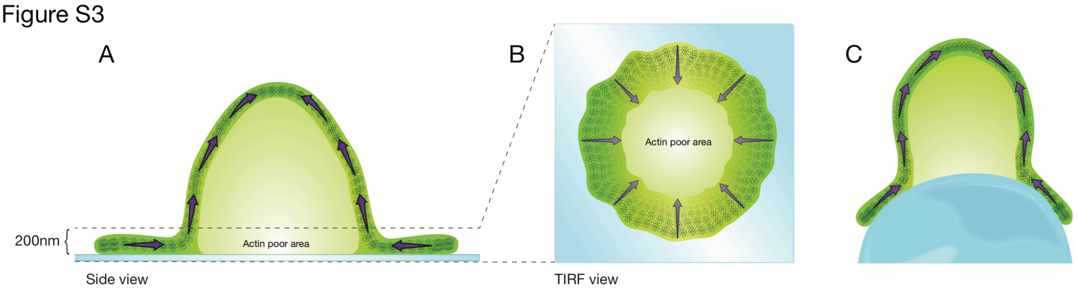

Figure S3: Actin flow in 2D versus 3D summary, related to Discussion. Cartoons to illustrate the flow of cortical actin in CTL cell synapses. (A) Side and (B) TIRF views of a rearward actin flow (purple arrows, away from the synapse) on a planar surface and (C) in a conjugate. By TIRF microscopy, in which only structures within 200nm of the coverslip are illuminated, actin would appear to move centripetally and then disappear. 3D imaging reveals that cortical actin continues to move rearward towards the back of the CTL. See also Movie S9.
