# Supplementary figures and images for: Actin Depletion Initiates Events Leading to Granule Secretion at the Immunological Synapse
Source: Immunity. 2015 May 19;42(5):864–76. doi: 10.1016/j.immuni.2015.04.013 (PMC4448150; doi:10.1016/j.immuni.2015.04.013)

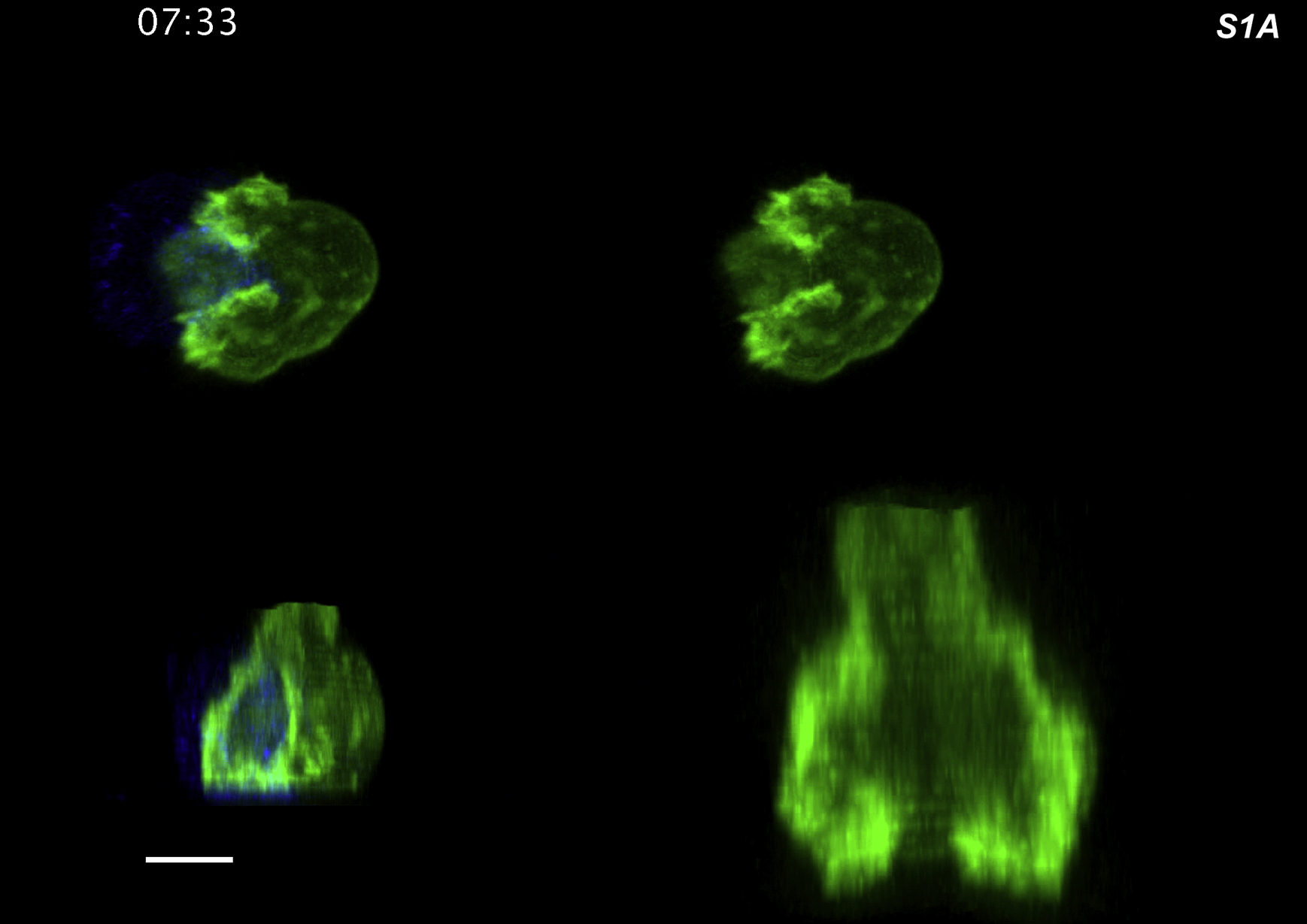

Supplement: Movie S1. 4D Actin Dynamics in CTLs — (A) Time-lapse maximum intensity projection of top-down view (upper left), side-on view (lower left), and en face view (lower right) of confocal sections of a CTL expressing Lifeact-mApple (green) as it interacts with a peptide-pulsed EL4-blue target. A top-down view of the 488-nm channel alone is shown in the upper right panel. Images were acquired on a 2-s interval. Scale bar represents 5 μm. (B) Time-lapse maximum intensity projection of confocal sections (left and center) and en face view (right) of a CTL expressing Lifeact-EGFP as it interacts with a peptide-pulsed EL4-blue target. The 488-nm channel alone is shown in the center panel. Images were acquired on a 20-s interval. Scale bars represent 5 μm (left and center panel) and 2 μm (right panel). Related to Figure 1. [file mmc2.jpg]

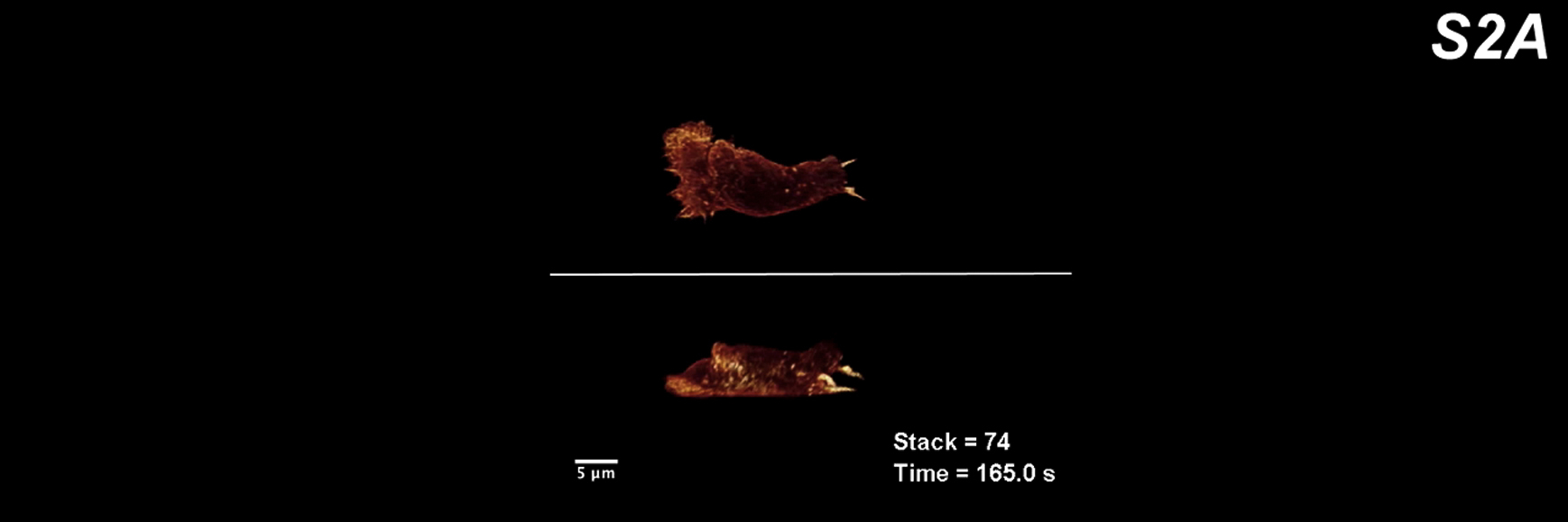

Supplement: Movie S2. Lattice Light-Sheet Microscopy of Actin in CTLs — (A) Time-lapse maximum intensity projections of lattice light-sheet images of a top-down (top panel) and side view (bottom panel) of a CTL expressing Lifeact-mEmerald as it migrates on ICAM-I-coated glass. Images were acquired on a 2.2-s interval. Scale bar represents 5 μm. (B) Time-lapse maximum intensity projection of lattice light-sheet images (left) and en face view (right) of a CTL expressing Lifeact-mEmerald (orange) as it interacts with a peptide-pulsed EL4-red target (cyan). Images showing the signal from the 488-nm channel in a single slice corresponding to the center of the synapse are shown in grayscale (center). Images were acquired on a 1.3-s interval. Scale bars represent 5 μm (left and center panels) and 3 μm (right panel). (C) Time-lapse maximum intensity projection of lattice light-sheet images (left) and en face view (right) of a CTL expressing Lifeact-mEmerald (orange) as it interacts with a peptide-pulsed EL4-red target (cyan). Images showing the signal from the 488-nm channel in a single slice corresponding to the center of the synapse are shown in grayscale (center). Images were acquired on a 1.68-s interval. (D) Grayscale image of cell in Movie S2C displays particles corresponding to visible actin structures that were tracked in three dimensions over time. Dragon tails showing the position of the particle over the previous five frames are color coded to show particle velocity. Images were acquired on a 1.68-s interval. Scale bar represents 5 μm. Related to Figure 2. [file mmc3.jpg]

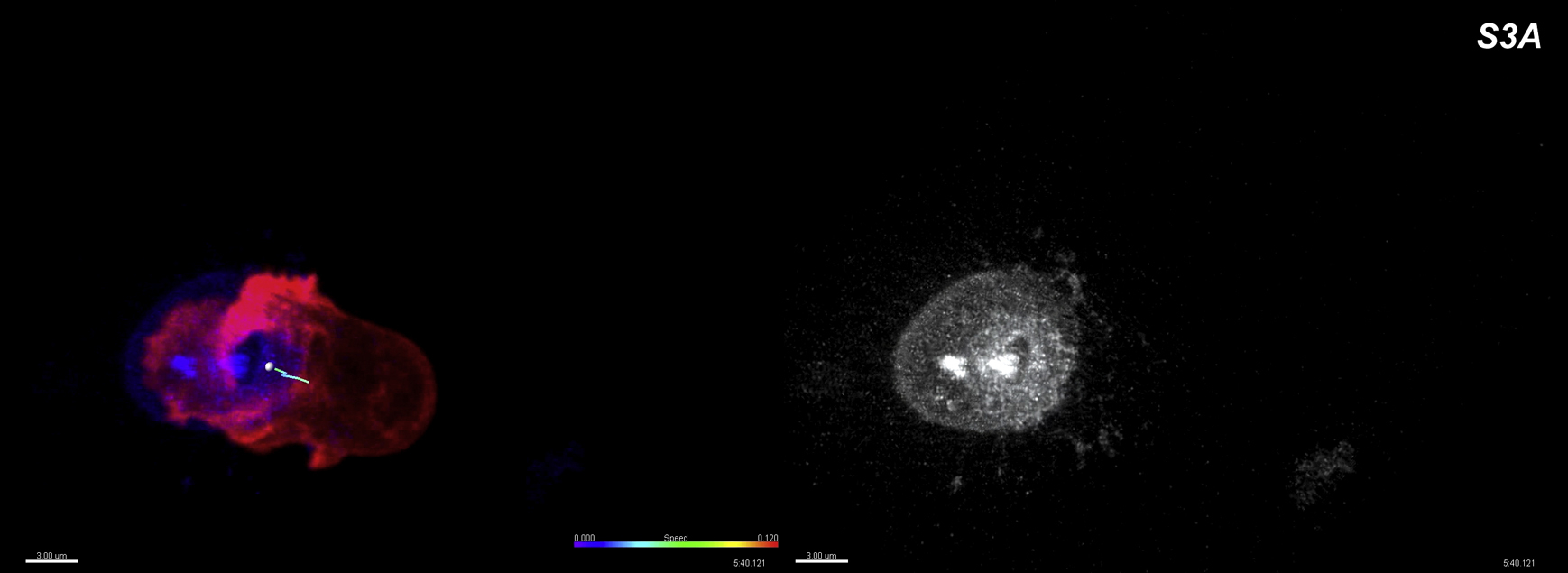

Supplement: Movie S3. Centrosome Migrates from the Uropod to the Center of the Actin-Deficient Zone — (A) Time-lapse maximum intensity projection images of confocal sections of a CTL expressing Lifeact-mApple and PACT-TagBFP (marked with a sphere) as it encounters an EL4-blue target cell. The track the centrosome takes to the target is represented by a dragon tail pseudocolored to indicate centrosome velocity. Signal from just the 405-nm channel is shown in the right panel. Images were acquired on a 20-s interval. Scale bar represents 3 μm. (B) Time-lapse maximum intensity projection images of confocal sections (left panel) and en face view (right panel) of a CTL expressing Lifeact-EGFP and PACT-mRFP as it encounters a target cell (red). Nuclei of both cells are labeled with Hoescht (blue). Images were acquired on a 20-s interval. Scale bars represent 5 μm (left and center panels) and 3 μm (right panel). 561- and 405-nm channels corresponding to the left panel are shown in the center panel. The scale of dynamic range is adjusted in the center panel to more accurately reflect the location of the centrosome signal. Related to Figure 3. [file mmc4.jpg]

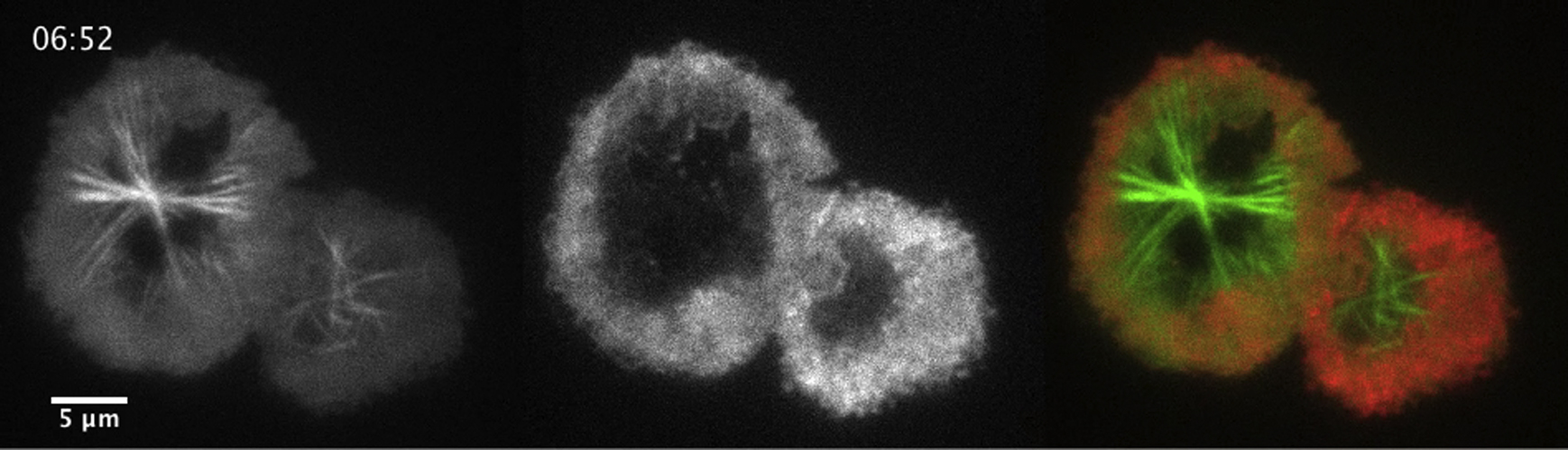

Supplement: Movie S4. TIRF Imaging of Actin and Microtubules in an Activated CTL — Time-lapse total internal reflection fluorescence (TIRF) images of a CTL expressing MAP Tau-EGFP (left panel) and Lifeact-mApple (center panel) as it interacts with glass that has been coated with an antibody against murine CD3ε. Images of merged channels are shown in the right panel. Images were acquired on a 2.4-s interval. Scale bar represents 5 μm. Related to Figure 4. [file mmc5.jpg]

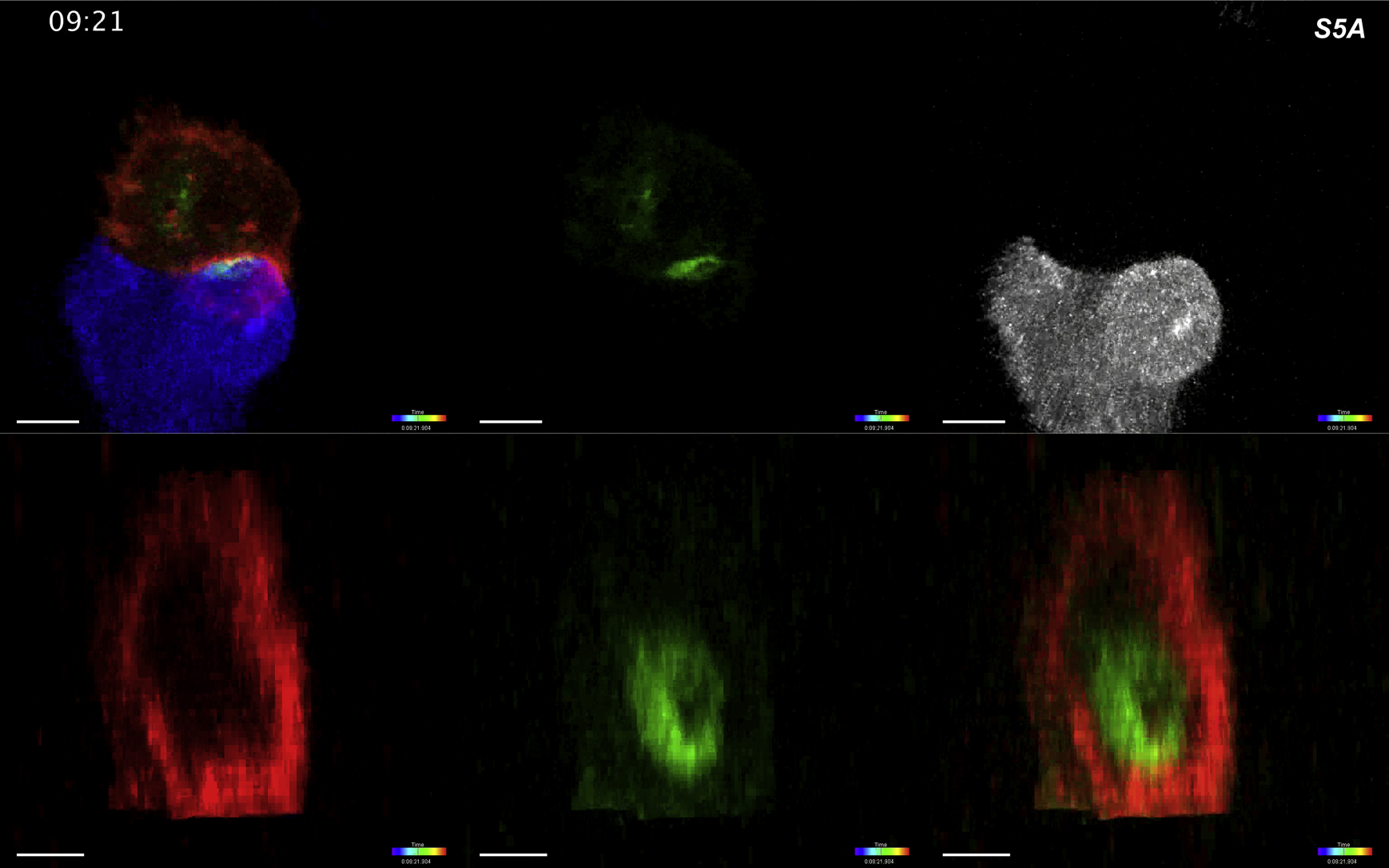

Supplement: Movie S5. 4D Dynamics of TCR in Relation to Cytoskeletal Elements — (A) Time-lapse maximum intensity projection images (upper panels) and reconstructed en face view (lower panels) of confocal sections taken as a CTL expressing CD3ζ-EGFP (green), Lifeact-mApple (red), and PACT-TagBFP encounters an EL4-blue target. Signal from the 405-nm channel is not displayed in the lower panels. A merged en face view is shown in the lower right panel. A white sphere marks the location of the PACT-TagBFP signal (upper left). Signal from the 488-nm channel alone is shown in the center panels. Signal from the 405-nm channel alone is displayed in the upper right panel to show centrosome location. Each displayed image corresponds to the same time point. Images were acquired on a 20-s interval. Scale bars represent 5 μm (upper panels) and 3 μm (lower panels). (B) Time-lapse maximum intensity projection images of a CTL expressing CD3ζ-EGFP and PACT-mRFP as it encounters an EL4-blue target cell. The 488- and 561-nm channels alone are shown in the center panel. An en face view of the CTL is shown in the right channel. Images were acquired on a 20-s interval. Scale bar represents 5 μm. Related to Figure 5. [file mmc6.jpg]

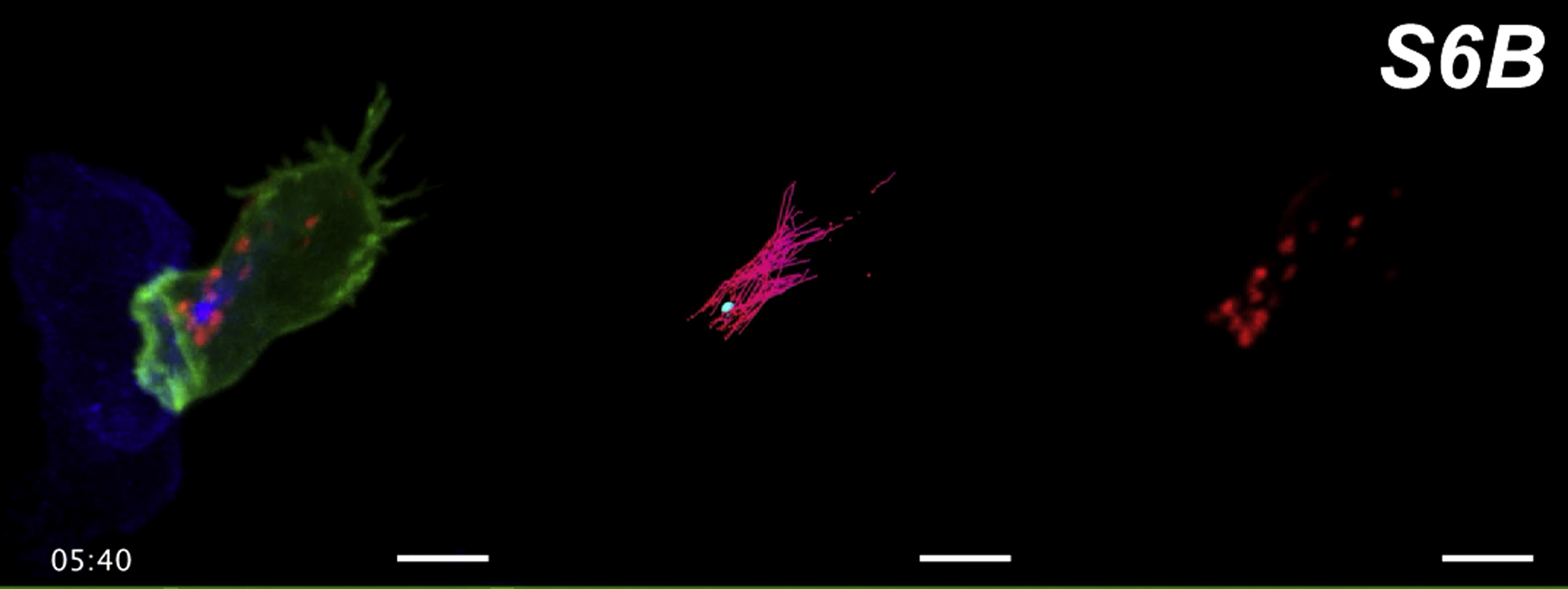

Supplement: Movie S6. 4D Dynamics of Cytolytic Granules in Relation to Cytoskeletal Elements — (A) Time-lapse maximum intensity projection images of confocal sections taken through a CTL expressing Lifeact-EGFP, CD63-mCherry, and PACT-TagBFP as it interacts with an EL4-blue target cell. All channels are shown in the left panel. The EGFP signal has been removed in the center panel, and a circle with a radius of 2 μm has been overlaid onto the centrosome as a reference. Signal from the 405-nm channel alone is displayed in the right panel to show centrosome location. Images were acquired on a 20-s interval. Scale bar represents 5 μm. (B) Time-lapse maximum intensity projection of confocal sections taken through a CTL expressing Lifeact-EGFP, CD63-mCherry, and MAPTau-TagBFP2 as it interacts with an EL4-blue target. A cyan sphere marks the location of the centrosome over time. Particles corresponding to the signal from the 561-nm channel (shown singly in the right panel) are tracked, and lines corresponding to the location of the particle for the previous four frames (dragon tails) are shown (center panel). Tracks are color coded to indicate relative time. Images were acquired on a 20-s interval. Scale bar represents 5 μm. Related to Figure 6A. [file mmc7.jpg]

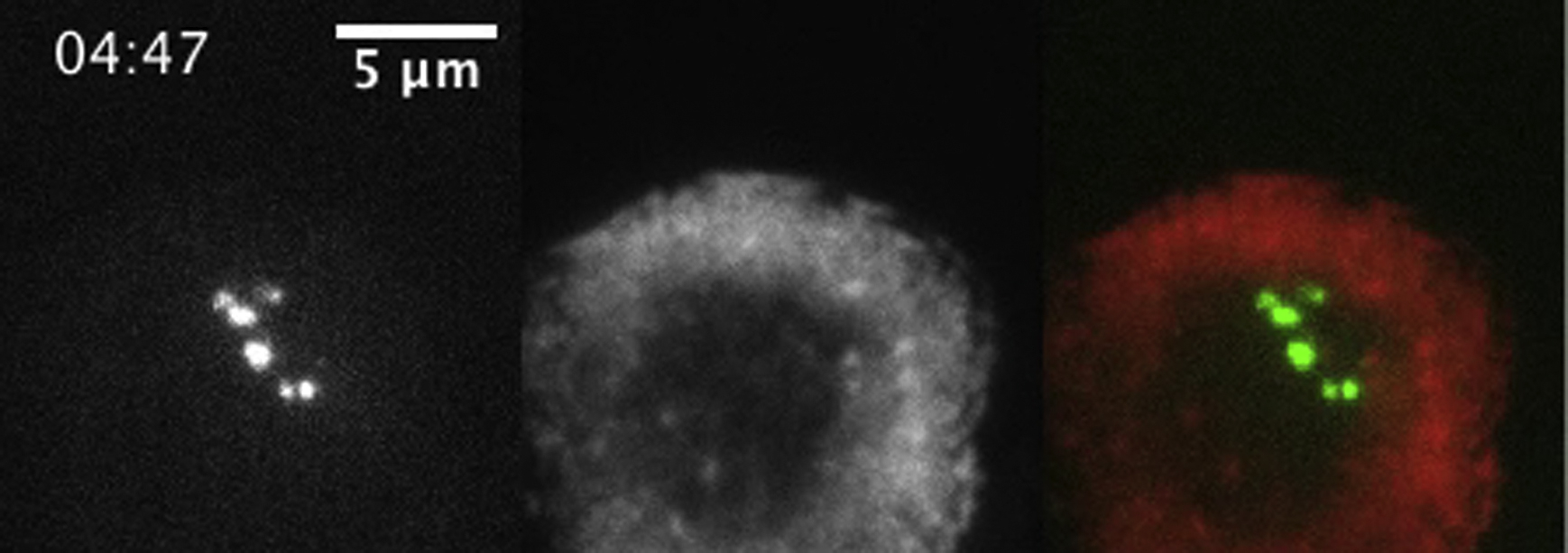

Supplement: Movie S7. Actin Dynamics and Cytolytic Granule Secretion Observed by TIRF Microscopy — TIRF movie of a CTL expressing Lamp1-EGFP (left) and Lifeact-mApple (center) as it interacts with glass that has been coated with an antibody against CD3ε. Images of merged channels are shown in the right frame. Two-color images were acquired every 2.4 s. Scale bar represents 5 μm. Related to Figure 7. [file mmc8.jpg]

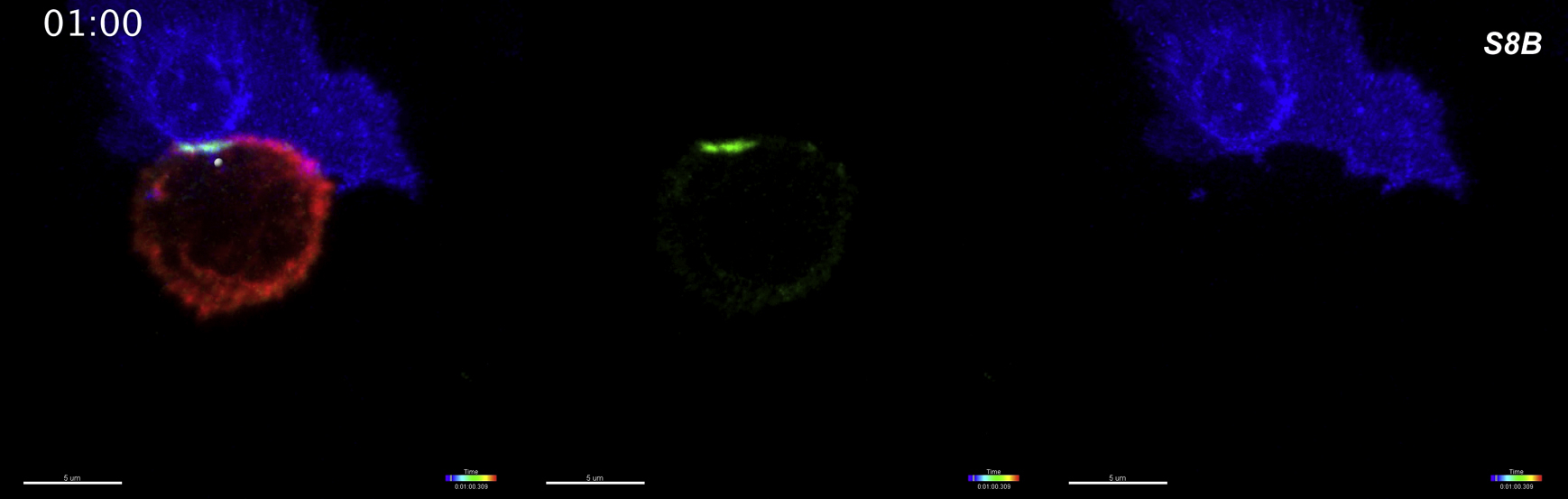

Supplement: Movie S8. Retraction of CTL from Target — (A) Time-lapse maximum intensity projection of confocal sections taken through a CTL expressing Lifeact-EGFP, CD63-mCherry, and MAPTau-TagBFP2 as it interacts with a MC57 target cell (blue). The location of the centrosome is marked with a white sphere. Signal from the 405-nm channel alone is displayed in the lower panel to show centrosome location. The location of the centrosome is tracked with a line color coded to indicate relative time. Images were acquired on a 15.6-s interval. Scale bar represents 5 μm. (B) Time-lapse maximum intensity projection of confocal sections taken through a CTL expressing CD3ζ-EGFP, Lifeact-mApple, and PACT-TagBFP (marked with a white sphere) as it interacts with an EL4-blue target. Single-channel time-lapse images from the 488-nm channel and the 405-nm channel are displayed in the center and right panels, respectively. Images were acquired on a 20-s interval. Scale bar represents 5 μm. Related to Figure 6. [file mmc9.jpg]

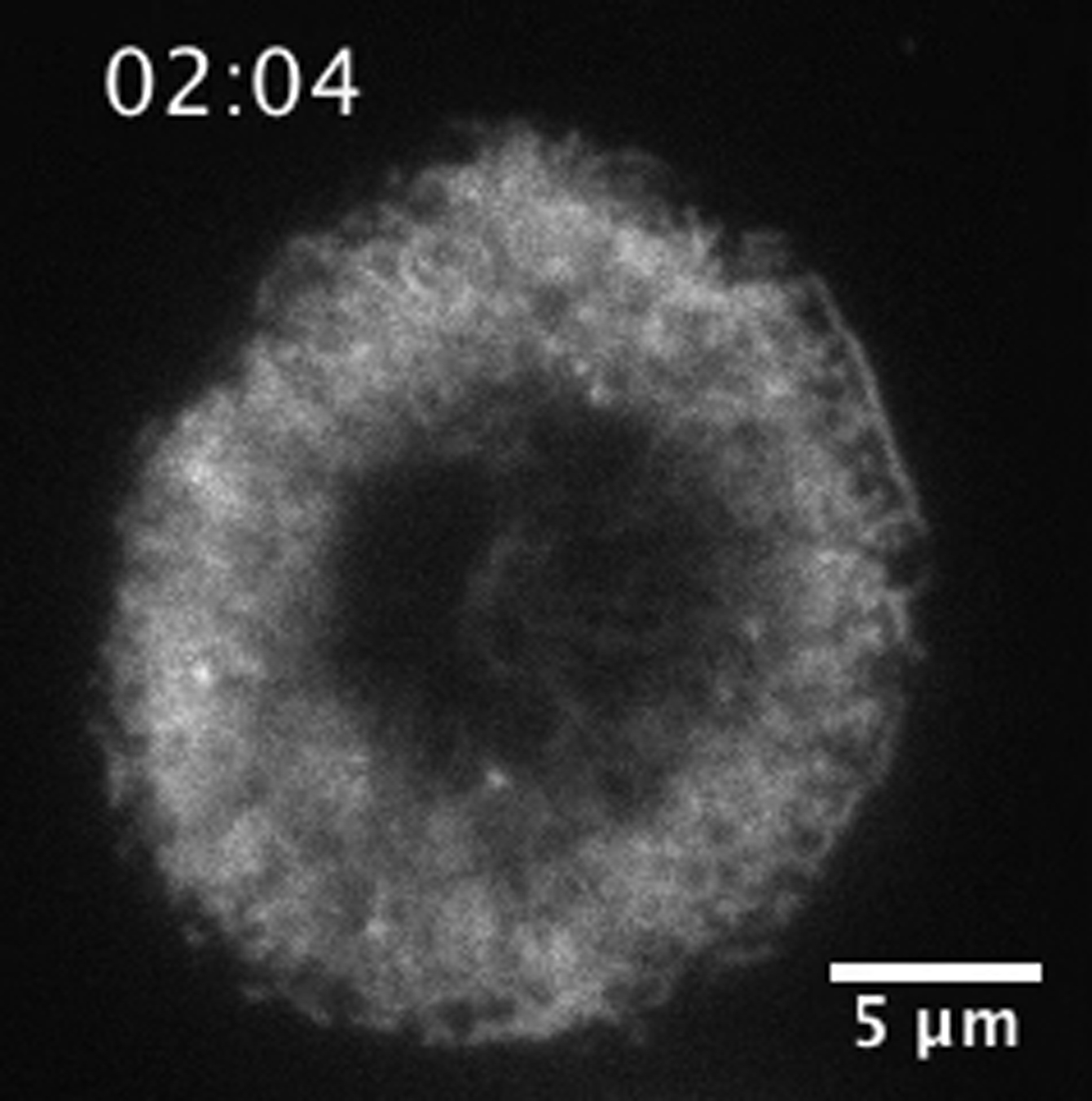

Supplement: Movie S9. Actin Flow Observed by TIRF Microscopy — TIRF movie of a CTL expressing Lifeact-EGFP as it interacts with anti-CD3ε-coated glass. Images were acquired every 2.3 s. Scale bar represents 5μm. Related to Figure 7. [file mmc10.jpg]
