# Supplementary material for: Actin Depletion Initiates Events Leading to Granule Secretion at the Immunological Synapse
Source: Immunity. 2015 May 19;42(5):864–76. doi: 10.1016/j.immuni.2015.04.013 (PMC4448150; doi:10.1016/j.immuni.2015.04.013)
Supplement: Document S2. Article plus Supplemental Information [file mmc11.pdf]

Immunity

Article

Actin Depletion Initiates Events Leading to Granule Secretion at the Immunological Synapse

Graphical Abstract

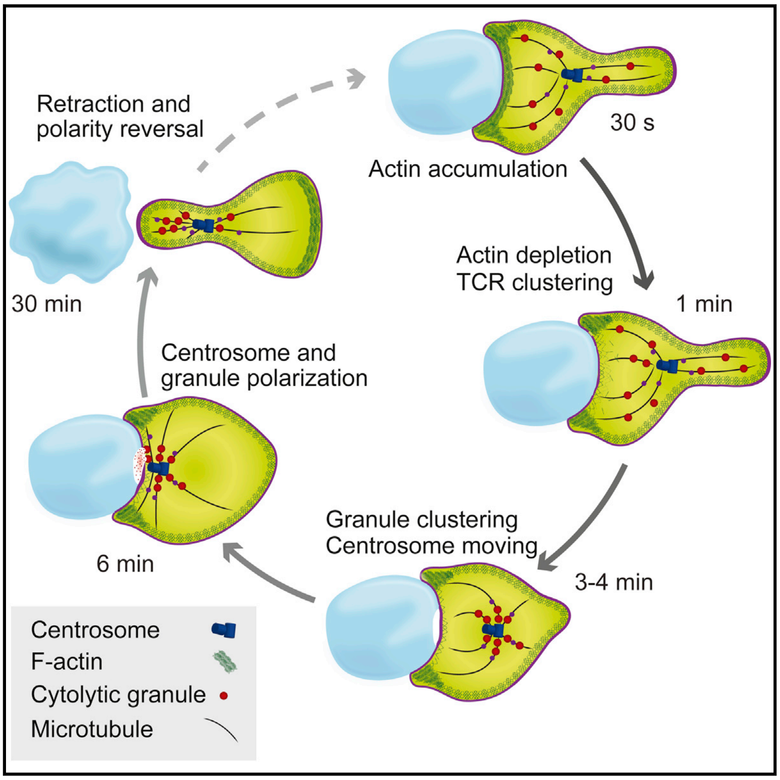

Highlights

- 4D imaging elucidates the order of events leading to secretion
- Actin depletion initiates events leading to centrosome polarization and secretion
- Lattice light-sheet imaging reveals a rearward flow of actin away from the synapse
- Both centrosome and granules are delivered to an area of membrane depleted of actin

Authors

Alex T. Ritter, Yukako Asano, ..., Jennifer Lippincott-Schwartz, Gillian M. Griffiths

Correspondence

lippincj@mail.nih.gov (J.L.-S.), gg305@cam.ac.uk (G.M.G.)

In Brief

The key sequence of events by which cytotoxic T lymphocytes establish an immunological synapse to kill target cells remains unclear. Griffiths and colleagues have resolved this by using high-resolution 4D imaging, revealing a critical role for actin reorganization in initiating centrosome polarization and granule secretion.

Ritter et al., 2015, Immunity *42*, 864–876
May 19, 2015 ©2015 Elsevier Inc.
http://dx.doi.org/10.1016/j.immuni.2015.04.013

Immunity
Article

Actin Depletion Initiates Events Leading to Granule Secretion at the Immunological Synapse

Alex T. Ritter,[1,2,8] Yukako Asano,[1,8] Jane C. Stinchcombe,[1] N.M.G. Dieckmann,[1] Bi-Chang Chen,[3,5] C. Gawden-Bone,[1] Schuyler van Engelenburg,[2,6] Wesley Legant,[3] Liang Gao,[3,7] Michael W. Davidson,[4] Eric Betzig,[3] Jennifer Lippincott-Schwartz,[2,9,*] and Gillian M. Griffiths[1,9,*]
[1]Cambridge Institute for Medical Research, University of Cambridge Biomedical Campus, Hills Road, Cambridge CB2 0XY, UK
[2]National Institute of Child Health and Disease, NIH, Bethesda, MD 20892, USA
[3]Howard Hughes Medical Institute, Janelia Farm Research Campus, 19700 Helix Drive, Ashburn, VA 20147, USA
[4]National High Magnetic Field Laboratory and Department of Biological Science, Florida State University, Tallahassee, FL 32304, USA
[5]Present address: Research Center for Applied Science, Academia Sinica, Taipei 11529, Taiwan
[6]Present address: Department of Biological Sciences, University of Denver, Denver, CO 80208-9010, USA
[7]Present address: Department of Chemistry, Stony Brook University, Stony Brook, NY 11794-3400, USA
[8]Co-first author
[9]Co-senior author
*Correspondence: lippincj@mail.nih.gov (J.L.-S.), gg305@cam.ac.uk (G.M.G.)
http://dx.doi.org/10.1016/j.immuni.2015.04.013

SUMMARY

Cytotoxic T lymphocytes (CTLs) use polarized secretion to rapidly destroy virally infected and tumor cells. To understand the temporal relationships between key events leading to secretion, we used high-resolution 4D imaging. CTLs approached targets with actin-rich projections at the leading edge, creating an initially actin-enriched contact with rearward-flowing actin. Within 1 min, cortical actin reduced across the synapse, T cell receptors (TCRs) clustered centrally to form the central supramolecular activation cluster (cSMAC), and centrosome polarization began. Granules clustered around the moving centrosome within 2.5 min and reached the synapse after 6 min. TCR-bearing intracellular vesicles were delivered to the cSMAC as the centrosome docked. We found that the centrosome and granules were delivered to an area of membrane with reduced cortical actin density and phospholipid PIP2. These data resolve the temporal order of events during synapse maturation in 4D and reveal a critical role for actin depletion in regulating secretion.

INTRODUCTION

The immunological synapse plays an important role in communication between immune cells by focusing signaling, secretion, and endocytosis at the point of contact between effector and antigen-presenting cells. The ability of immune cells to direct secretion very precisely is key for accurate delivery of signals between cells. This is particularly important for cytolytic cells, such as cytotoxic T lymphocytes (CTLs) and natural killer (NK) cells, which destroy the targets they recognize through polarized secretion of cytolytic granules containing perforin and granzymes. Accurate polarized secretion ensures that CTLs destroy only the cell recognized and not neighboring bystanders.

Upon T cell receptor (TCR)-mediated recognition of a target cell, receptors that are involved in target recognition and adhesion organize to form the central and peripheral supramolecular activation clusters (cSMAC and pSMAC, respectively; Monks et al., 1998), which are surrounded by a distal ring enriched with actin (dSMAC) (Freiberg et al., 2002; Sims et al., 2007; Stinchcombe et al., 2001b). Secretion occurs into a specialized secretory domain, which lies next to the cSMAC and within the pSMAC. A secretory cleft, which appears as an indentation in the membrane of the target cell, lies opposite the secretory domain (Stinchcombe et al., 2001a; Stinchcombe et al., 2001b). Precise secretion is ensured by the positioning of the centrosome, which contacts the plasma membrane at the cSMAC (Stinchcombe et al., 2006). This allows cytolytic granules, moving along microtubules in a dynein-mediated minus-end direction, to be delivered accurately to the plasma membrane at the immunological synapse.

Confocal, super-resolution, and electron microscopy have provided high-resolution images of secretion from CTLs and NK cells, but piecing together the order of events that lead to secretion from images of fixed cells can be misleading. Likewise, imaging of live cells has a number of inherent problems because T cells are famously small and round and "never sit still for a picture" (Poenie et al., 2004). Consequently, the resolution of live images has been limited. In order to gain the resolution required for determining the formation of the immunological synapse, many studies have taken advantage of imaging artificial planar synapses of T cells formed on antibody-coated glass coverslips or supported lipid bilayers loaded with ligands that stimulate the T cell (reviewed in Balagopalan et al., 2011). These artificial synapses lend themselves to imaging by total internal reflection fluorescence (TIRF) microscopy, which provides improved resolution and sensitivity within the TIRF field. However, TIRF imaging is only able to provide information about structures within 100–200 nm of the coverslip, which represents 1%–2% of the entire T cell, and does not capture events in the rest of the cell.

 ©2015 Elsevier Inc.

Trying to piece together the order of events leading to secretion from different studies produces a confusing picture. Confocal imaging on fixed CTL-target conjugates has shown a correlation between docking of the centrosome at the plasma membrane and clearance of actin from the synapse (Stinchcombe et al., 2006). However, super-resolution imaging of NK cell synapses has revealed granules associated with a meshwork of actin across the synapse (Brown et al., 2011; Rak et al., 2011), suggesting a role for the actin meshwork in granule release. The mechanism of cSMAC formation is also unclear, given that TCR microcluster delivery to the cSMAC is thought to be driven by a centripetal inward flow of actin across the synapse, coupled with dynein-driven transport along microtubules (Hashimoto-Tane et al., 2011; Kaizuka et al., 2007; Varma et al., 2006). Because dynein mediates minus-end movement along microtubules toward the centrosome, this model suggests that the centrosome reaches the synapse before the cSMAC forms (Babich and Burkhardt, 2011). This raises questions as to whether TCR accumulates at the cSMAC before or after the centrosome reaches the plasma membrane.

To resolve these issues and provide a clear picture of the order of events leading up to secretion at the immunological synapse, we made use of both spinning-disc confocal microscopy and lattice light-sheet microscopy (Chen et al., 2014) to image multiple components of CTLs in three dimensions from the earliest point of T cell interaction with a target through to secretion. Our results identified actin depletion across the synapse as an initiating event leading to cSMAC formation in two stages and delivery of both the centrosome and granules to an area of membrane with significantly reduced cortical actin. Four-dimensional (4D) super-resolution imaging revealed a continual rearward flow of actin when the synapse was formed. Our results highlight a critical role for actin depletion in the regulation of secretion.

RESULTS

Actin Accumulates and Clears from the Synapse within the First Minute of Target Cell Interaction

We first mapped the timing of actin reorganization as the synapse forms between CTLs and targets. Throughout this study, we made use of CTLs generated from OT-I TCR transgenic mice, because they provide a uniform TCR-mediated recognition of target cells, and peptide-pulsed EL4 target cells stably expressing plasma membrane markers fused to either TagBFP2 (EL4-blue) or TagRFP (EL4-red). CTLs expressing EGFP or mApple fused to the F-actin-binding peptide Lifeact (Riedl et al., 2008) were imaged with a spinning-disc microscope as they migrated on glass coated with intercellular adhesion molecule 1 (ICAM-I) and interacted with targets. Every 20 s, the microscope captured a 3D stack of 12–14 2D images spaced at 0.8 μm over the entire volume of the cell.

Motile primary CTLs assumed the stereotypical tadpole shape with an actin-rich leading edge and a uropod protruding from the rear of the cell (time = 0:00; Figure 1A). Imaging in 3D over time allowed us to monitor the overall morphology of each CTL as it transitioned from a solitary, motile cell to form a conjugate. We found that the uropod, which is present in migratory cells, gradually retracted into the cell body through the course of target interaction, as observed in earlier phase-microscopy studies (Yannelli et al., 1986). The uropod rounded up into the cell body within 3–5 min (average = 243 ± 91 s, n = 15 cells) after the initial CTL-target contact (Figure 1A; Movie S1).

As the CTL contacted the target cell, polymerized actin accumulated across the synapse, as seen in a single confocal section taken through the conjugate at the center of the contact interface (Figures 1Bii–1Biv; Movie S1). This rapidly changed within the next minute as we observed a gradual drop in the density of cortical actin within the center of the synapse, whereas the perimeter of the CTL-target interface remained densely labeled with F-actin. The intensity of Lifeact labeling in the center of the synapse decreased below the levels seen in the rest of the cell body (n = 15 cells; Figure 1B), on average 52 s (±10 s) after the initial target contact. We found that the intensity of Lifeact inside the dSMAC decreased to ~20% of that in the rest of the cell body after 2 min of interaction.

Lattice Light-Sheet Microscopy Reveals a Retrograde Flow of Cortical Actin during Synapse Formation

By imaging CTLs after target recognition, we found that actin underwent a dramatic reorganization within the first 2 min of synapse formation. The acquisition rate of our spinning-disc microscope was limited to one volume per 20 s, so we sought a way to better spatially and temporally resolve this rapid actin reorganization at the synapse. Lattice light-sheet microscopy enables very rapid, high-resolution imaging of 3D volumes over time (Chen et al., 2014). It can be thought of as an improvement on Bessel beam plane-illumination microscopy (Gao et al., 2012), in which a massively parallel set of beams replaces a single beam to further increase speed and reduce phototoxicity. Using this method, we were able to image a full 3D volume consisting of 131 2D planes of CTL-target conjugates in two colors every 1.3 s for hundreds of time points, giving us 3-fold finer optical sectioning and greater than 15-fold improvement in temporal resolution over spinning-disc confocal microscopy.

Imaging migrating CTLs with the lattice light-sheet microscope revealed actin-based ruffles and projections emerging from the leading edge and moving toward the uropod of the CTLs (n = 9 cells; Figure 2A; Movie S2); these were similar to the actin-based membrane ruffling and rearward actin flow observed in other migrating cells (Burnette et al., 2011; Giannone et al., 2007). When the CTLs encountered the target, projections at the leading edge formed an initial accumulation of actin across the CTL-target interface, as illustrated in Figure 2B and Movie S2 (16.6 s). In this movie, actin polymerization focused at the periphery of the synapse by 33 s after target cell contact, and actin projections were generated exclusively around the periphery by 41 s. By this time, the density of cortical actin within the synapse dropped below that in the rest of the cell (Figure 2Bii). Strikingly, the same rearward movement of actin structures observed in migratory T cells was also seen in conjugates and persisted throughout imaging (Figure 2C; Movie S2). Tracking visible actin structures in 3D over time revealed a mean speed of 0.31 μm/s (±0.089 μm/s; Figure 2D; Movie S6).

The information that the extremely high temporal resolution gained by lattice light-sheet microscope revealed about migrating and conjugating CTLs was impossible to see at the

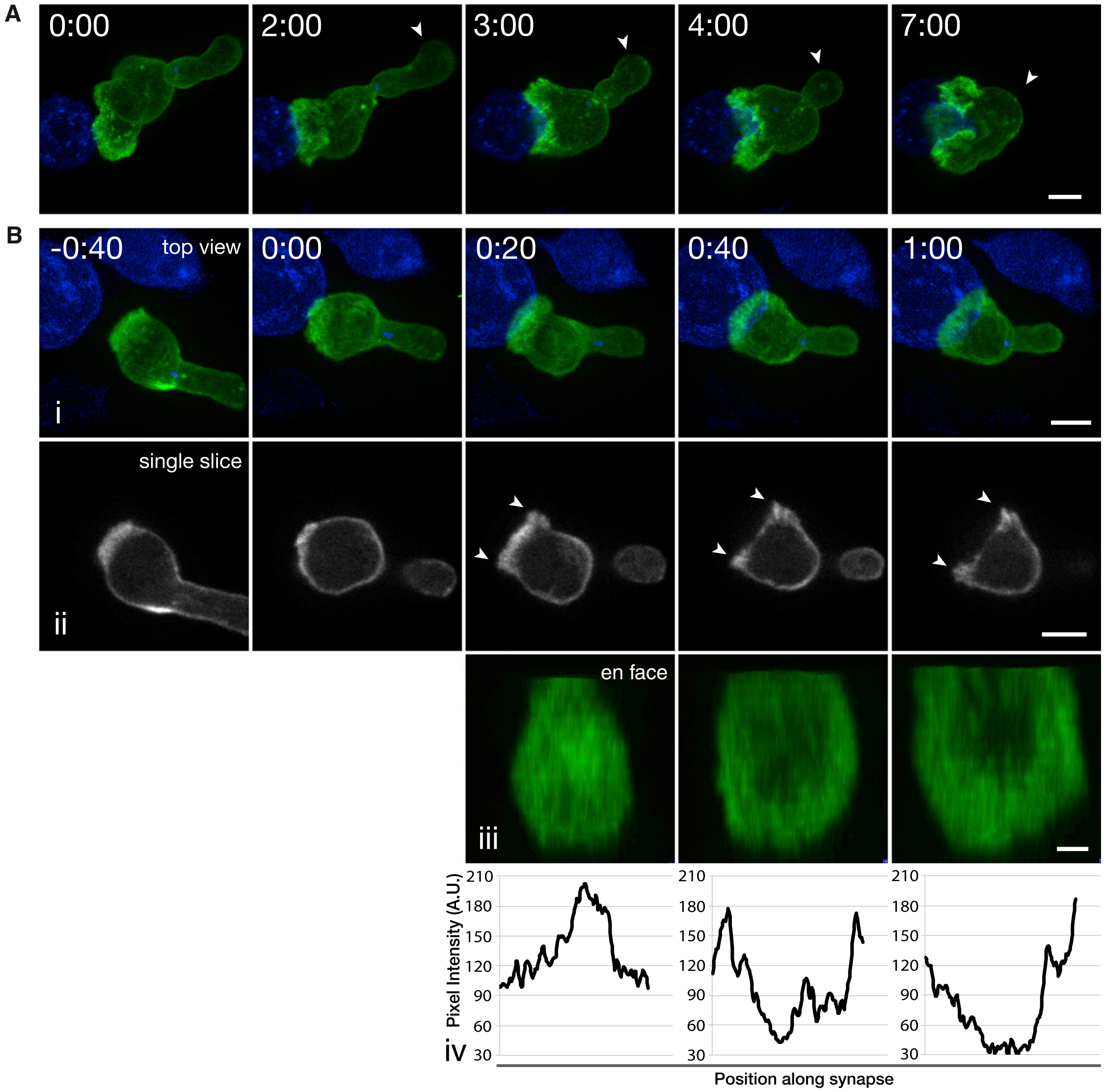

Figure 1. Actin Initially Accumulates at the Synapse and Quickly Clears to Form an F-Actin-Depleted Region Surrounded by an Actin-Rich Lamella

(A) Maximum intensity projection (MIP) of a Lifeact-mApple-expressing CTL (green) interacting with a target (blue). Arrows point out the uropod as it retracts into the cell body over the course of target interaction (n = 15 cells, 8 independent experiments, i.e., from different mice). Scale bar represents 5 μm.

(B) Time lapse of (i) MIP of confocal sections, (ii) a single confocal slice corresponding to the center of the synapse from the 488-nm channel, and (iii) en face view of a Lifeact-EGFP-expressing CTL interacting with an EL4 target cell expressing a farnesylated TagBFP2 (blue) (n = 15 cells, 3 independent experiments). Time 0:00 indicates the point of first contact with the target. (iv) Fluorescence-intensity plots quantify actin density along the synapse between the arrowheads shown in (ii). Scale bars represent 5 μm (i and ii) and 2 μm (iii).

Time is shown as min:s. See also Movie S1.

slower acquisition rate of a spinning-disc confocal microscope. These data reveal that migratory CTLs and those forming conjugates have remarkably similar actin dynamics, including projections forming at the leading edge and actin flowing rearward toward the uropod. The major changes that occur upon synapse formation are the loss of cortical actin density from the center of the synapse and the relocation of lamellipodial actin polymerization to the dSMAC at the perimeter of the synapse. This all happens within 1 min after the T cell encounters the target.

Centrosome Polarization Begins within the First Minute as Cortical Actin Density Decreases across the Synapse

Initial studies on fixed samples showed a correlation between the docking of the centrosome at the plasma membrane and the clearance of actin from the synapse, suggesting that the two events might be linked (Stinchcombe et al., 2006). Therefore, we asked how the timing of centrosome polarization is related to the decrease in cortical actin density across the synapse. We tracked the route of the centrosome from the uropod to the synapse in CTLs expressing fluorescently tagged Lifeact

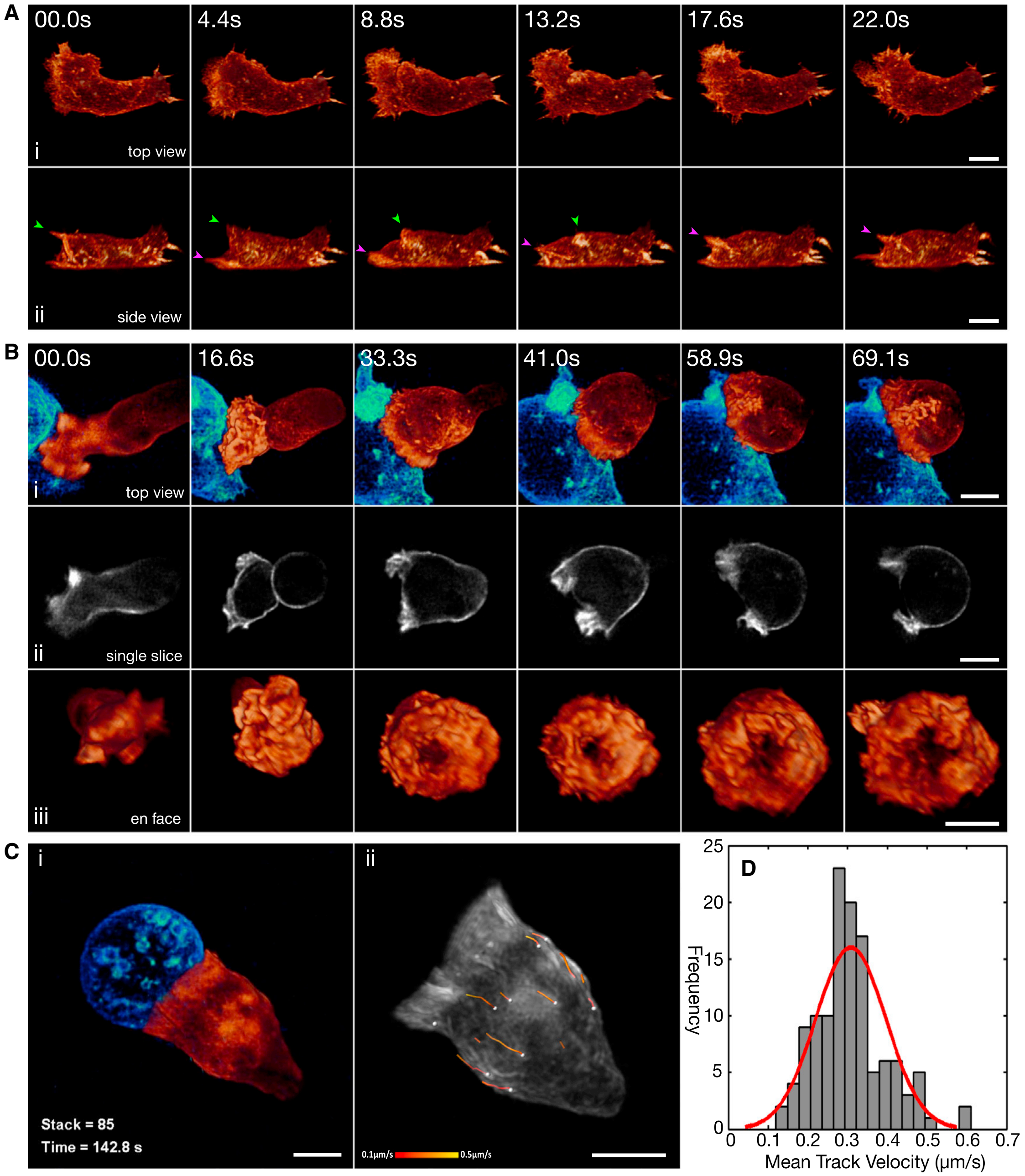

Figure 2. Lattice Light-Sheet Microscopy Reveals Actin Dynamics in Conjugating T Cells

(A) Time-lapse MIP of coherent structured light-sheet images of (i) top views and (ii) side views of a migrating CTL expressing Lifeact-mEmerald. Green and magenta arrows highlight ruffles that translate up the dorsal surface of the cell (n = 9 cells, 3 independent experiments).

(B) Time-lapse MIP of (i) lattice light-sheet images, (ii) a single slice corresponding to the center of the synapse from the 488-nm channel, and (iii) en face view of a Lifeact-mEmerald-expressing CTL (orange) interacting with an EL4-red target (cyan) (n = 10 cells, 3 independent experiments).

(C) (i) MIP of a Lifeact-mEmerald-expressing CTL (orange) interacting with a target cell (cyan). (ii) Particles corresponding to visible actin structures were tracked in three dimensions over time. Dragon tails showing particle positions over the previous five frames are color coded to show particle velocity.

(D) Mean velocity for each track was determined and collated into a histogram with a line of fit (red) (n = 123 tracks over 8 cells from 3 independent experiments). Scale bars represent 5 μm. Time is shown as min:s. See also Movie S2.

and PACT domain (which labeled the centrosome) (Figure 3A; Movie S3). Centrosome migration from the uropod began within the first minute simultaneously with the reorganization of actin across the synapse (Figures 3A and 3B). It took an average of 6.1 min (366 ± 97.1 s; n = 16 cells, 9 independent experiments) for the centrosome to translocate from the uropod to the synapse upon CTL-target contact with an average velocity of 2.5 μm/min (±0.92 μm/min; n = 54 cells,

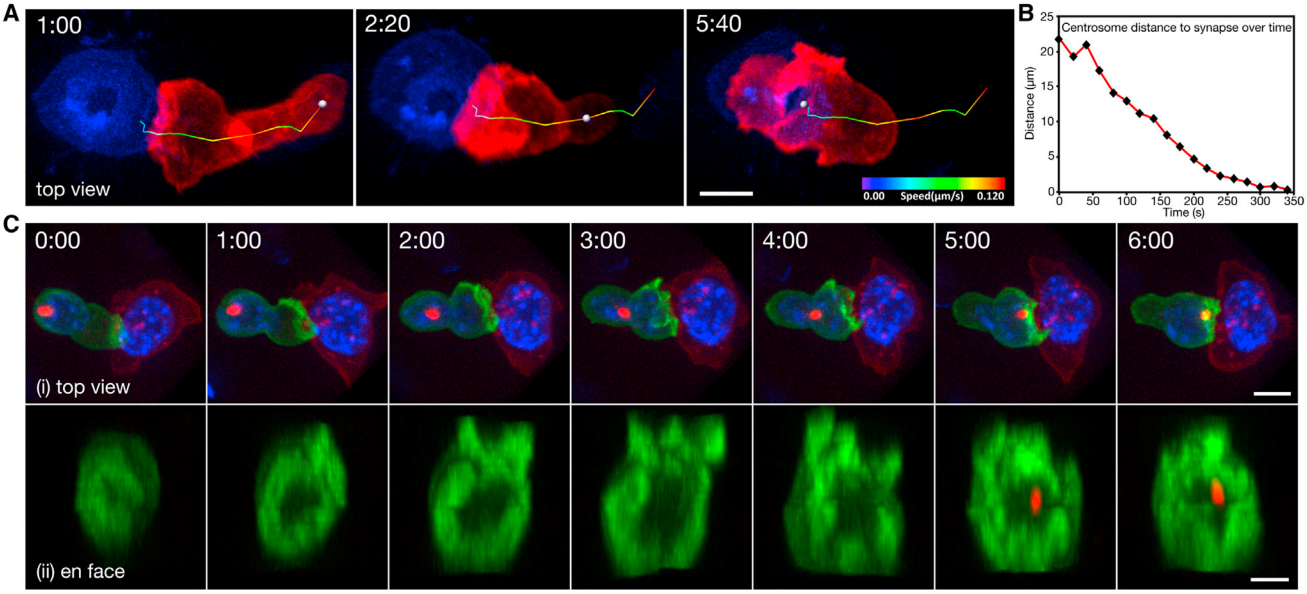

Figure 3. Centrosome Migrates from the Uropod to the Center of the Actin-Deficient Zone

(A) Time-lapse MIP images of a CTL expressing Lifeact-mApple and PACT-TagBFP (marked with a sphere) as it encountered an EL4-blue target cell. The track the centrosome took to the target is represented by a line pseudocolored to indicate particle velocity (n = 16 cells, 9 independent experiments). Scale bar represents 5 μm.

(B) Graph of centrosome distance to synapse versus time for the dataset in (A).

(C) Time lapse of (i) MIP images or (ii) en face view of a CTL expressing Lifeact-EGFP and PACT-mRFP as it encountered a target cell (red). The nuclei of both cells are labeled with Hoescht (blue). Scale bars represent 5 μm (i) and 3 μm (ii).

Time is shown as min:s. See also Movie S3.

31 independent experiments). The velocity of the centrosome varied on its path to the synapse and reached speeds of up to 5 μm/min.

We asked whether actin depletion across the synapse persists over the 6 min required for centrosome polarization by imaging both actin and the centrosome in polarizing CTLs. We found that actin cortical density at the synapse remained low throughout the course of centrosome translocation across the cell (n = 16 cells; Figure 3; Movie S3). In Figure 3C, actin depletion was already apparent in the center of the synapse at t = 0 and persisted for >6 min as the centrosome came within the depth of the en face view (2 μm) at 5–6 min. These results show that centrosome polarization begins as the changes in actin organization are initiated at the synapse. The centrosome takes 6 min to reach the synapse and contacts the plasma membrane after cortical actin density at the synapse is minimal.

The Centrosome Docks at the Plasma Membrane when Cortical Actin Is Reduced

Given that previous studies used artificial planar synapses and phalloidin to reveal a meshwork of actin across the synapse in NK cells (Brown et al., 2011; Rak et al., 2011), we examined this with a similar system, including glass coverslips coated with anti-CD3ε or non-stimulatory anti-CD25. Using phalloidin to detect F-actin, we acquired images by using structured illumination microscopy (SIM), which provides twice as much image resolution in three dimensions as do conventional confocal or widefield imaging approaches (Gustafsson, 2000).

SIM images of CTLs plated on anti-CD3ε-coated glass showed that when the centrosome, which is the microtubule organizing center (MTOC) in CTLs (and the point from which microtubules radiate out), was visible (within 200 nm of the coverslip), it was centered within the region of reduced cortical actin and surrounded by a ring of densely labeled actin (n = 21 cells; Figure 4A). Conversely, CTLs plated on non-stimulatory anti-CD25 maintained a dense cortical actin layer, which separated the microtubules from the plasma membrane (n = 24 cells; Figure 4B). Lateral (xz) and axial (yz) sections through the cells revealed that the cortical actin was significantly less dense at the interface of CTLs plated on anti-CD3ε than at the interface of CTLs plated on anti-CD25. In TCR-stimulated CTLs, where the cortical actin density was decreased, the microtubule network was able to come very close (200 nm) to the contact interface.

In order to obtain dynamic images of actin and microtubule networks on a planar synapse, we used TIRF microscopy to image mature CTLs expressing Lifeact-mApple and MAPTau-EGFP as they interacted with a coverslip coated with anti-CD3ε. We found that, as for CD4 cells (Bunnell et al., 2001), CTLs rapidly polymerized actin and spread on an activating surface (n = 17 cells; Figure 4Cii). A decrease in the density of actin in the center of the synapse was accompanied by the appearance of the centrosome (MTOC) and microtubules in the TIRF field (Movie S4). The centrosome remained centralized relative to the surrounding actin, and microtubules appeared to lie parallel to the synapse while the cortical actin density was reduced (2.5–7.5 min; Figure 4C). By 15 min, actin recovered into the deficient region and was accompanied by a loss of MAPTau-EGFP

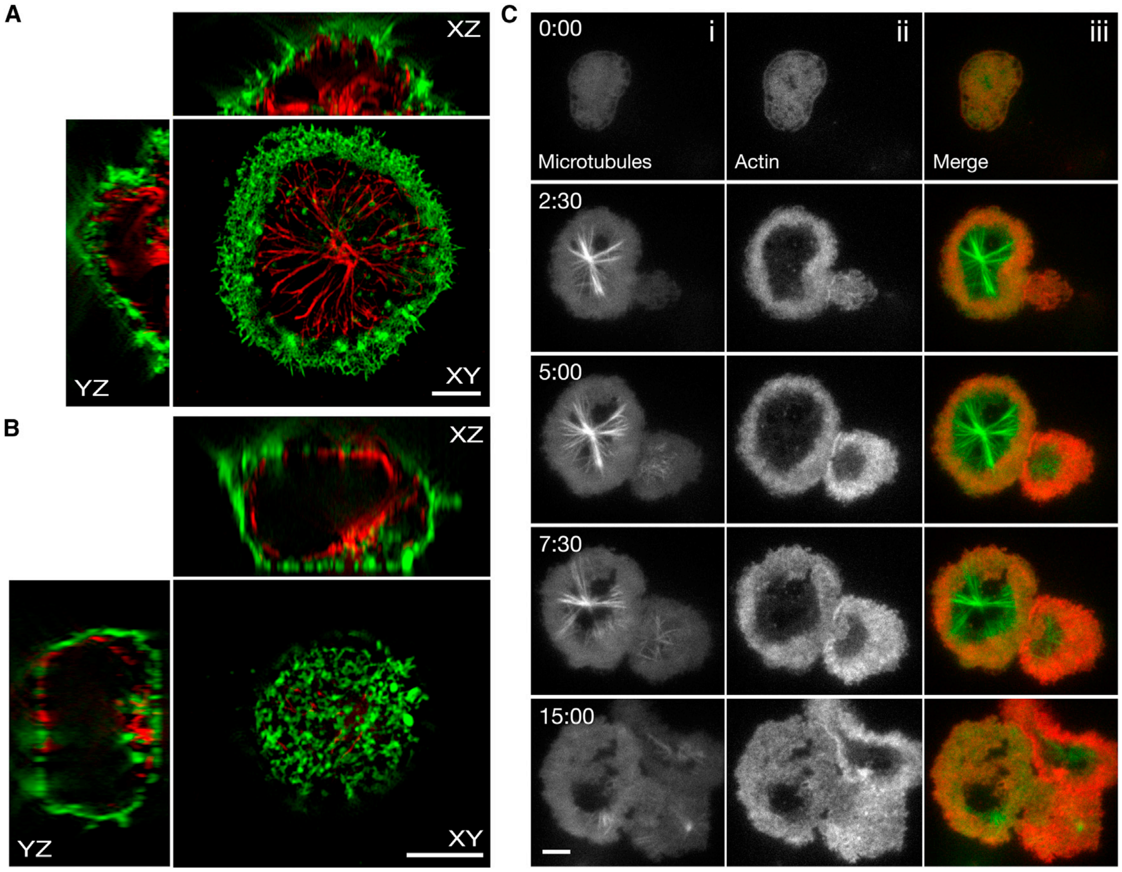

Figure 4. Cortical Actin Is Reduced at the Synapse when the Centrosome Is Docked

(A and B) 3D structured-illumination images of a CTL that was fixed while interacting with glass coated with an antibody against CD3ε (A: n = 21 cells, 3 independent experiments) or CD25 (B: n = 24 cells, 3 independent experiments) and stained with phalloidin-Alexa 488 (green) and an antibody against α-tubulin (red). The "XY" panels are MIP images of the bottom 0.22 μm of the cell. The "XZ" and "YZ" panels are 0.3-μm-thick slices through the center of the cell in each respective dimension. Scale bars represent 5 μm.

(C) TIRF images of (i) a CTL expressing MAP Tau-EGFP (n = 17 cells, 5 independent experiments) and (ii) a CTL expressing Lifeact-mApple as they interacted with glass coated with an antibody against CD3ε. Images of merged channels are shown in (iii). Scale bar represents 3 μm.

See also Movie S4.

signal, indicating that microtubules were moving out of the TIRF field as actin accumulated across the synapse (15 min; Figure 4C). Taken together, these results show that the centrosome and microtubules localize at the synapse only when cortical actin density is diminished.

cSMAC Formation Occurs in Two Stages, and the Centrosome Controls Vesicular TCR Delivery

Given that centrosome polarization is triggered by TCR signaling (reviewed in Huse et al., 2013), we asked how the timing of TCR clustering to form a cSMAC is related to actin reorganization and centrosome polarization. Using CTLs transiently expressing the zeta chain of TCR tagged with EGFP (CD3ζ-EGFP), we followed the movement of the TCR as migrating CTLs encountered targets relative to actin dynamics (Lifeact-mApple) and centrosome movement (PACT-TagBFP) (Figure 5; Movie S5). CD3ζ-EGFP localized predominantly to the plasma membrane of CTLs. In 83% of migrating cells, the plasma-membrane-localized component of CD3ζ-EGFP preferentially localized to the cortex of the uropod. An additional population of CD3ζ-EGFP localized to intracellular vesicles in 84% of cells (n = 42 cells; Figure 5A).

Within 2 min of contacting the target cell, CD3ζ-EGFP in CTLs coalesced to form a cSMAC within the central area of reduced actin density. This is clearly shown in the en face views of the synapse (Figures 5Bii–5Biv; Movie S5). We analyzed the accumulation of the CD3ζ-EGFP by drawing traces along the synapse in single-plane confocal sections and compared fluorescence intensity of CD3ζ-EGFP to that of actin (Figure 5Bv). These showed that the fluorescence intensity of CD3ζ-EGFP at the cSMAC remained at the same level between 2 and 4 min but increased by 10 min.

We found that the cSMAC was visible before the centrosome docked at the synapse in 91% of imaged conjugates (n = 32 cells), such that the cSMAC was detected within ~2 min, and the centrosome docked after ~6 min (Figures 5B and 5C; Movie S5). The initial accumulation of CD3ζ-EGFP at 2 min most likely occurred by lateral translocation, and it became visible as it clustered. However, an additional pool of CD3ζ-EGFP, associated

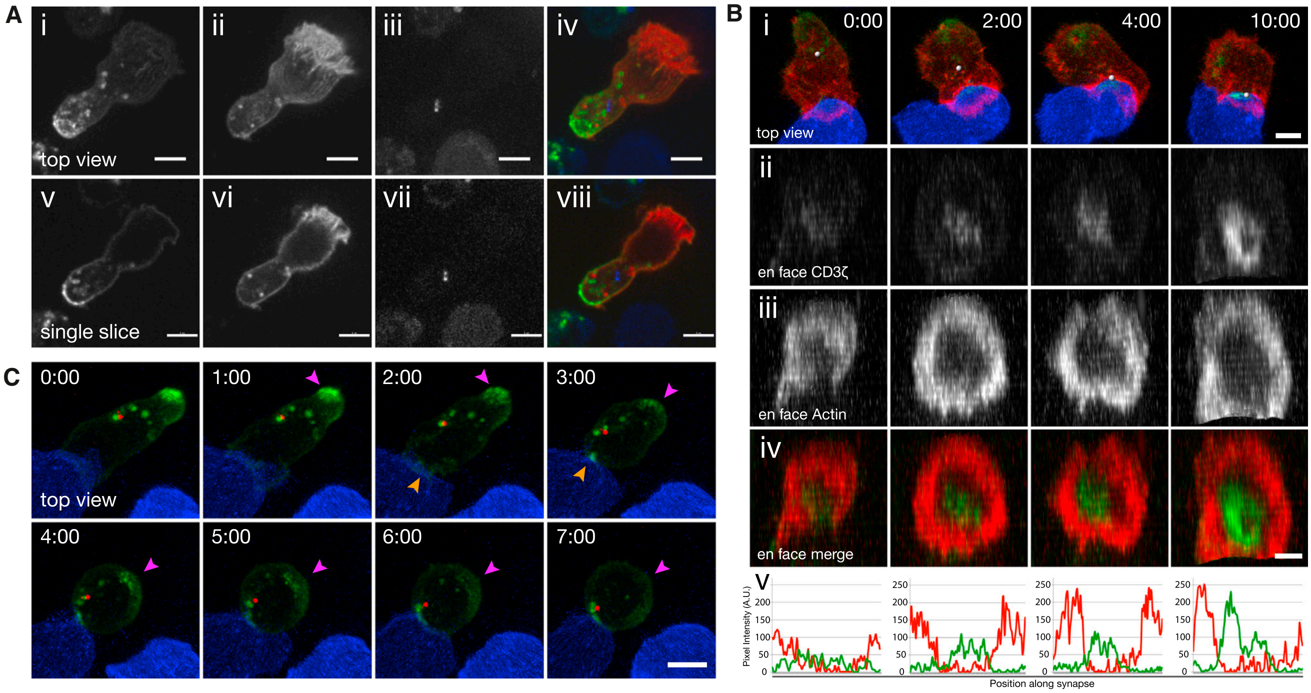

Figure 5. cSMAC Formation Occurs in Two Stages, before and after Centrosome Polarization

(A) MIP (i–iv) and single confocal slice (v–viii) of a migratory CTL expressing CD3ζ-EGFP (i and v), Lifeact-mApple (ii and vi), or PACT-TagBFP (iii and vii). Images of merged channels are also shown (iv and vii). Scale bars represent 5 μm.

(B) Time-lapse MIP images (i) and reconstructed en face view (ii–iv) of confocal sections taken as a CTL expressing PACT-TagBFP (i), CD3ζ-EGFP (ii), or Lifeact-mApple (iii) encountered a target cell (blue). A merged en face view is shown in (iv). A white sphere marks the location of the PACT-TagBFP signal in (i). (v) Fluorescence-intensity plots of a line scan across the synapse of a single z slice at the center of the synapse show EGFP and mApple signal in green and red, respectively. Scale bars represent 5 μm (i) and 3 μm (ii–iv).

(C) Time-lapse MIP images of a CTL expressing CD3ζ-EGFP and PACT-mRFP as it encountered a target cell (blue). Magenta arrows highlight EGFP signal disappearing from the distal part of the cell, and orange arrows indicate EGFP signal accumulating at the cell-cell interface. Scale bar represents 5 μm. Time is shown as min:s. (n = 32 cells, 15 independent experiments). See also Movie S5.

with intracellular vesicles, moved to the synapse with the centrosome and reached the cSMAC ~6 min after contact (Figures 5B and 5C; Movie S5). This vesicular pool of CD3ζ-EGFP provides an explanation for the increased intensity of the cSMAC at 10 min (Figures 5Biv and 5Bv).

These results reveal that the cSMAC forms in two stages in relation to the centrosome. Initially, the TCR translocates laterally at the synapse before the centrosome docks; then, a second wave of TCR located on intracellular vesicles is delivered to the cSMAC as the centrosome reaches the synapse.

Cytolytic Granule Delivery Occurs in Two Distinct Steps

We next asked when lytic granule secretion occurs in relation to these events. CTLs expressing Lifeact-EGFP and PACT-TagBFP together with CD63-mCherry, which marks the cytolytic granules, were imaged as they formed conjugates with peptide-pulsed EL4-blue targets (Figure 6A). We assessed the spatial relationship between lytic granules and the centrosome by determining 3D coordinate positions for each granule and the centrosome at each time point by using Imaris software (Bitplane). We calculated the mean distance of the granules to the centrosome over time and found that upon CTL recognition of the target cell, granules rapidly collapsed toward the centrosome. The mean granule-to-centrosome distance reached its minimum value ~2.5 min after the CTL recognized its target, whereas the centrosome reached the plasma membrane at the synapse on average 5.8 min after target recognition (n = 9 cells; Figure 6B). These values indicate that the granules cluster at the centrosome prior to arrival at the synapse. This clustering caused a bolus of granules to be delivered to the synapse simultaneously with centrosome polarization to an area of membrane with reduced levels of cortical actin (Figure 6C; Movie S6).

We also tracked granule dynamics throughout the time that the centrosome was adjacent to the synapse. We found that granules remained tightly associated with the centrosome as it polarized to the synapse. Upon centrosome contact with the plasma membrane, the mean granule-centrosome distance increased slightly (n = 9 cells; Figure 6B), such that 85% of the granules remained polarized toward the target cell around the centrosome for >14 min after contact was made. Unexpectedly, 15% of granules were observed to cycle from the centrosome back into the cell body before returning to the synapse (Figure 6C; Movie S6). However, because we used the lysosomal membrane protein CD63 to mark granules, it is possible that not all CD63 compartments were perforin-containing cytolytic granules.

These results reveal that granule polarization occurs in two distinct steps after CTL recognition of the target cell. TCR

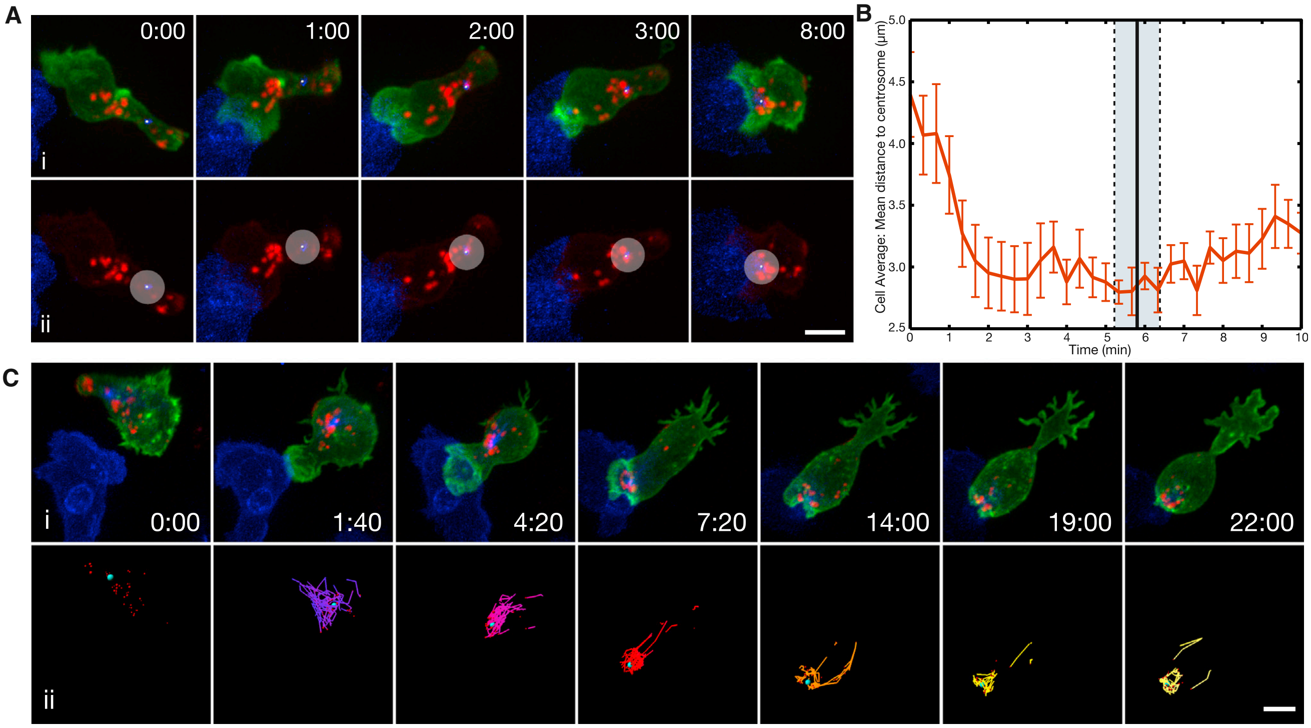

Figure 6. Granules Cluster at the Centrosome before It Arrives at the Synapse

(A) Time-lapse MIP images of a CTL expressing Lifeact-EGFP, CD63-mCherry, and PACT-TagBFP as it interacted with a target cell (blue). All channels are shown in (i). The EGFP signal has been removed in (ii), and a circle with a radius of 2 μm has been overlaid onto the centrosome as a reference.

(B) Graph of the average distance between granules and the centrosome over time. The black vertical line indicates the average time it took the centrosome to reach the synapse in sample cells, and the gray box indicates the SE. Error bars represent the SE at each time point (n = 9 cells, 3 independent experiments).

(C) (i) Time-lapse MIP images of a CTL expressing Lifeact-EGFP, CD63-mCherry, and MAPTau-TagBFP2 as it interacted with a target cell (blue) (n = 9 cells, 3 independent experiments). (ii) Particles localized to the mCherry signal from (i) are shown. A cyan sphere marks the location of the centrosome over time. Particles corresponding to granules are tracked, and tracks corresponding to the location of the particle for the previous four frames (dragon tails) are shown. Tracks are color coded according to time.

Time is shown as min:s. Scale bars represent 5 μm. See also Movies S6 and S8 and Figure S1.

signaling triggers an initial clustering of granules toward the centrosome within the first 2.5 min after target cell recognition. Then, granule polarization occurs together with centrosome polarization, and the granules reach the synapse at the same time as the centrosome. These results suggest that the granules are delivered to an area of reduced cortical actin.

Cytolytic Granule Secretion Occurs in an Area of Reduced Actin Density

In order to examine granule release, we imaged CTLs expressing Lifeact-mApple and Lamp1-EGFP by using TIRF microscopy on anti-CD3-coated glass coverslips. This provided the best spatial and temporal resolution (images were captured every ~2 s) within 200 nm of the plasma membrane, allowing us to see the rapid release of granules in relation to actin. The dynamics of actin in CTLs interacting with antigen-coated glass mirrored those observed in CTLs engaging targets. Actin first accumulated at the synapse as the T cell initially spread at the activation site. This was followed by a reduction in cortical actin density in the center of the synapse (Figure 7A; Movie S7). After the thinning of the actin cortex, lytic granules labeled with Lamp1-EGFP were observed to enter the TIRF field, indicating that they were within 200 nm of the plasma membrane. Shortly after this, degranulation occurred, as evidenced by the diffusion of the Lamp1-EGFP signal into the plasma membrane (time = 5:30; Figure 7A; Movie S7). The dynamics of actin in relation to granule secretion can be easily viewed in Figure 7B, in which a kymograph of these data depicts the fluorescence intensity under a 5-pixel-thick line overlaid on the cell over time. The location of the line in one frame is shown in the bottom right panel of Figure 7A. These results show very clearly that degranulation occurs in areas of reduced actin density and that actin density only recovers across the synapse once secretion has occurred (n = 14 cells; Movie S7).

Actin Depletion Correlates with Reduced Phosphatidyl Inositide-4,5-Bisphosphate at the Synapse

We asked whether other changes occur within the membrane where cortical actin is depleted. Given that phosphatidyl inositide-4,5-bisphosphate (PIP2) plays a role in recruitment of cortical actin to membranes (Botelho et al., 2000; Janmey and Lindberg, 2004; Raucher et al., 2000), we examined PIP2 localization across the synapse when cortical actin levels were depleted (Figure 7C). We found that levels of PIP2 at the synapse were lower than those of a non-specific marker for the plasma membrane (farnesyl-EGFP; n = 150 cells, 3 independent experiments). Reduced PIP2 was correlated with cortical actin

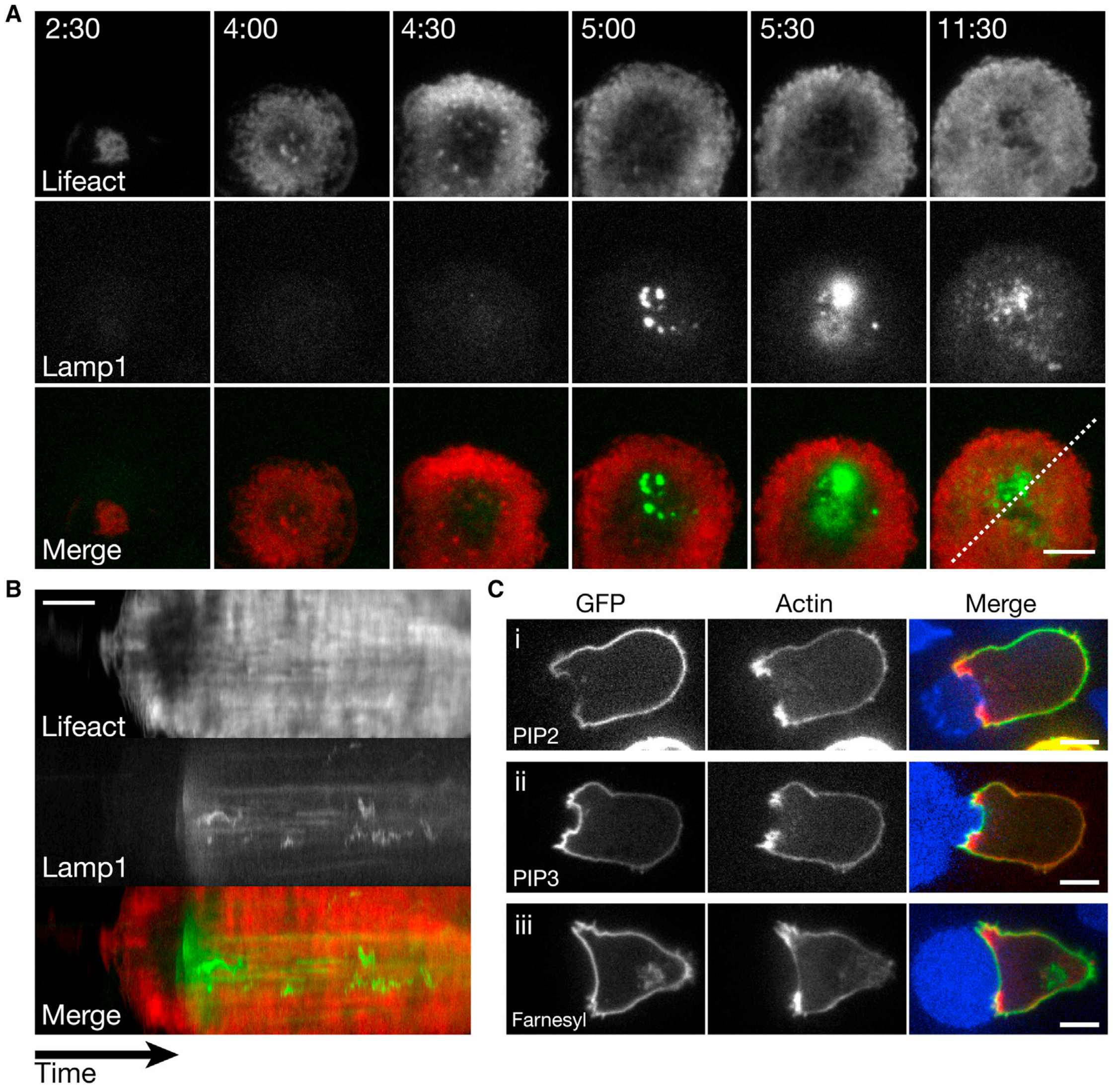

Figure 7. Lytic Granule Secretion Occurs after Cortical Actin Reduction

(A)Time-lapse TIRF images of a CTL expressing Lifeact-mApple (top) or Lamp1-EGFP (middle) as it interacted with glass coated with an antibody against CD3ε (n = 14 cells, 5 independent experiments). Images of merged channels are shown in the bottom frames. Scale bar represents 5 μm.

(B) Kymograph of the movie shown in (A). Fluorescence intensity under a 5-pixel-thick line is measured over time. The position of the line (dashed white line) is shown in the lower right panel of (A). Signal corresponding to Lifeact-mApple is shown on top, signal corresponding to Lamp1-EGFP is shown in the middle, and merged signal is shown on the bottom. Scale bar represents 2 min.

(C) Single confocal slices of a CTL expressing Lifeact-mApple along with the lipid binding domain of (i) Tubby (n = 190 cells, 4 independent experiments), (ii) AKT (n = 57 cells, 2 independent experiments), or (iii) a farnesylation sequence fused to EGFP (n = 150 cells, 3 independent experiments) as it interacted with an EL4-blue target cell. Scale bars represents 5 μm.

See also Movies S7 and S9.

(n = 190 cells, 4 independent experiments). In agreement with previous studies (Costello et al., 2002; Le Floc'h et al., 2013), we found that PIP3 (detected by AKT-EGFP) accumulated at the synapse (n = 57 cells, 2 independent experiments).

Polarity Reversal during CTL Retraction from the Target

In this study, we focused primarily on imaging the early events in CTL interaction with target cells (Figure 6). However, in some cases, we were also able to capture CTLs as they retracted from target cells after killing (n = 13 cells; Figure S1; Movie S8), as shown by an MC57 target cell that rounded up upon killing, allowing us to document the termination of the synapse. We noted that actin polymerization initiated at the rear of the CTL before retraction from the target (17–21 min; Movie S8). By 29 min, the centrosome started to retract from the synapse as the CTL detached from the target. By 31 min, the CTL migrated away from the target, and the retracted centrosome localized to the newly formed uropod of the cell (Figure S1). As the CTL departed, the synapse transformed into the uropod such that CD3ζ remained clustered and appeared as an accumulation at

the uropod (Figure S1). Pieces of target cell membrane (blue) were pulled toward the departing CTL (Movie S8), suggesting that significant forces were generated, and puncta of CD3ζ also transferred to the target (Movie S8).

These data suggest that as CTLs detach from the synapse, they reverse their polarity by initiating an actin-rich lamellipodia at the distal pole. The centrosome, granules, and clustered TCR are then localized in the newly forming uropod as the CTL pulls away from its target. In this way, the polarity of the CTL is elegantly reversed.

DISCUSSION

In this study, we used high-resolution 3D time-lapse multi-color imaging to define the changes in cell architecture as CTLs transitioned from migration to secretion at the immunological synapse. Using techniques that image the entire cell volume, we have revealed a clear order of events leading to secretion (Figure S2). Our studies used high spatial and temporal resolution to show the dynamics of actin in CTLs interacting with live target cells in three dimensions, providing important new insights into the mechanisms involved in CTL secretion. (1) We observed that migratory CTLs produced lamellipodial projections and a rearward flow of actin structures as they engaged targets. (2) We found that the cSMAC formed in two stages, first by lateral translocation of the TCR within the plasma membrane (at 1 min) and subsequently by vesicular delivery of the intracellular TCR as the centrosome reached the synapse (at 6 min). (3) Finally, we found that both the centrosome and granules docked in an area of low actin density, and a meshwork of actin appeared only after secretion had occurred.

A number of controversies surround formation of the immunological synapse and the site of granule secretion. Questions have arisen as to (1) whether cSMAC formation requires centripetal actin flow or dynein-mediated movement along microtubules, (2) how the roles of the plasma membrane and intracellular pools of TCR might differ, and (3) whether actin clears or remains as a fine meshwork through which granules are secreted (Brown et al., 2011; Rak et al., 2011). Our findings provide a new 4D model of the events leading to secretion by revealing the temporal order of events across the whole cell from migration to secretion (Figure S2).

Our studies show that in OT-I CTLs recognizing ovalbumin (OVA)-pulsed EL4 cells, the initial contact between a CTL and its target occurs via actin-rich protrusions from the CTL, and these form a wall of densely polymerized actin as the CTL contacts the target. Within 30 s after contact, a dramatic reorganization of actin takes place as actin polymerization focuses at the periphery of the synapse and the density of actin at the center of the synapse decreases. By 1 min after contact, cortical actin density at the center of the synapse is significantly lower than that of the rest of the CTL body. As actin diminishes centrally, the TCR coalesces by lateral translocation in the plasma membrane into the center of the cleared ring. A clearly identifiable cSMAC is visible within 2 min after initial contact. Meanwhile, in the rear of the cell, the cytolytic granules rapidly cluster toward the centrosome within the next 30 s as the centrosome begins to polarize toward the synapse and reach the plasma membrane on average 6 min after contact between the CTL and the target. As the centrosome reaches the synapse, intracellular vesicles containing the TCR are also delivered to the cSMAC, and the intensity of TCR at the cSMAC increases. We found that degranulation occurs next to the centrosome in an area of low actin density, which recovers across the synapse once secretion has occurred.

Once the centrosome reaches the plasma membrane, other changes occur. Within 2 min of centrosome docking, the tight clustering of granules around the centrosome relaxes slightly. Both the centrosome and the granules can then remain polarized at the synapse membrane for up to 20 min, during which time the cell shape at the distal pole extends to form a lamellipodium. The centrosome and granules eventually retract into the new uropod, which forms at the interface with the dying target as the CTL departs, and the polarity of the CTL is elegantly reversed. The whole cycle is complete within approximately 30 min, and the CTL can move off to find subsequent targets. The timing of CTL-mediated arrest and killing is consistent with reports made from in vivo studies showing 9.8 min between arrest and lysis (Mempel et al., 2006).

A number of important new observations emerge from our study of the intracellular events leading to killing. First, our imaging clearly shows that the rearward flow of actin structures in migrating cells continues once the synapse is formed. Our observations support the suggestion that the synapse resembles a radially symmetric migratory cell in which the dSMAC (characterized by protrusive actin polymerization and the presence of the Arp2/3 complex) is equivalent to the lamellipodium, and the pSMAC (enriched with integrins and myosin IIa) is analogous to the lamellum (Babich et al., 2012; Dustin, 2005; Yi et al., 2012).

Our observation of a rearward flow of actin from the synapse to the distal pole seems to contrast with our own observations and other 2D planar-surface studies (Ilani et al., 2009; Kaizuka et al., 2007), which showed an inward centripetal flow of actin. However, because T cells spread and flatten extensively on planar surfaces (Balagopalan et al., 2011; Beemiller et al., 2012; Bunnell et al., 2001; Varma et al., 2006), actin-rich rings of $\sim$5 μm can form at the periphery (Babich et al., 2012), and a rearward actin flow seen in 3D (Figure 2; Movie S2) will correspond to an inward centripetal flow of actin at the periphery of the synapse when it is viewed in 2D on a planar surface (Figure 7; Figure S3; Movie S8). We found that the rearward rate of actin flow (0.31 μm/s) is the same as the rate of centripetal actin flow observed in TIRF studies of the synapse (Babich et al., 2012; Kaizuka et al., 2007), thus supporting the idea that centripetal inward flow is the 2D view of the rearward flow seen in 3D. Our study suggests a very simple model in which the rearward flow of actin structures seen in migratory CTLs is maintained when the synapse forms.

The second important finding of this study is that the cSMAC forms in two stages, whereby the centrosome docks after the initial TCR clustering and controls the secondary delivery of vesicular TCR. Although early studies showed that the rapid coalescence of the TCR to form the cSMAC within the first 2 min of contact was dependent on actin reorganization (Campi et al., 2005; Kaizuka et al., 2007; Varma et al., 2006), a later study showed that TCR migration to the cSMAC was dependent on dynein-mediated movement of the TCR along microtubules

(Hashimoto-Tane et al., 2011). Our data resolve the apparent discrepancies between these earlier reports by revealing the timing of two waves of TCR delivery to the cSMAC: first (within 2 min), the TCR is laterally translocated within the plasma membrane while actin reorganizes across the synapse, and second (4 min later), a pool of intracellular TCR is delivered within vesicles when the centrosome docks. The second wave of intracellular TCR delivery would be mediated by dynein given that both centrosome and vesicle movement require dynein activity (reviewed in Huse et al., 2013), thus providing an explanation for the previously observed dynein-mediated TCR delivery (Hashimoto-Tane et al., 2011). Endocytic pools of TCR have been proposed to both deliver TCR to and recycle TCR from the cSMAC (Das et al., 2004; Soares et al., 2013). Furthermore, the endocytic pool of CD3ζ has been shown to be tyrosine phosphorylated (Yudushkin and Vale, 2010). Our results show that the cSMAC forms before the centrosome docks, consistent with the idea that the TCR pool could be modulated by the delivery or recycling of TCR upon arrival of the centrosome at the synapse.

The third important finding is that cortical actin density across the synapse decreases below levels elsewhere on the cell and that this decreased density persists until granule secretion has occurred. A concurrent loss of PIP2 from the membrane supports the idea that recruitment of cortical actin is reduced in this area, given that PIP2 plays a role in recruiting cortical actin (Botelho et al., 2000; Janmey and Lindberg, 2004; Raucher et al., 2000). These changes are consistent with our previous studies showing a change in membrane architecture from actin-rich interdigitations to a flattened interface as the synapse forms (Jenkins et al., 2014). Super-resolution studies on NK cell synapses in fixed cells have revealed a fine actin meshwork across the synapse, suggesting that granules might be secreted through holes in this meshwork (Brown et al., 2011; Rak et al., 2011). In some cell types, including melanocytes and mast cells, granules are first tethered to cortical actin, which then regulates secretion (Porat-Shliom et al., 2013; Wollman and Meyer, 2012). However, in CTLs, in which granules are delivered by centrosomal docking at the plasma membrane, this is not the case. By monitoring actin and lytic granule dynamics over time, our study supports a model in which secretion occurs at a time when cortical actin is greatly reduced at the point of delivery.

Other intriguing observations emerge from our study of the order of events leading to secretion. Whereas previous studies have documented both the clustering of granules at the centrosome without polarization (Beal et al., 2009) and centrosome polarization in the absence of granule clustering (Jenkins et al., 2009) in response to different stimuli, the reason for these discrepancies has remained unclear. The present study reveals that granule clustering around the centrosome precedes centrosome polarization toward the synapse, suggesting that discrete signals control granule clustering and centrosome movement.

This study produces a new, 4D model of the immunological synapse, providing a temporally resolved model of the events that lead to polarized secretion at the immunological synapse initiated by TCR-triggered depletion of actin across the synapse.

EXPERIMENTAL PROCEDURES

DNA Constructs

Constructs used in this paper were generated as follows. For CD3ζ-EGFP, full-length murine CD247 amplified by PCR from OT-I CTL cDNA was cloned into the XhoI and SalI sites of pEGFP-C1 (Clontech); for PACT-TagBFP, PACT (Gillingham and Munro, 2000) was cloned into the BglII and XbaI sites of pTagBFP-C (Evrogen); for EGFP-MAPTau, MAPTau was subcloned into the XhoI and BamHI sites of pEGFP-N1; and for TagRFP-MEM-pMig, TagRFP was substituted in EYFP-MEM (Clontech) and Farnesyl-TagBFP2-pMig, which were subcloned into a pMig-R1 retroviral vector. Lifeact-mApple, Lifeact-mEmerald, mTagBFP2-MAPTau-N10, and mTagBFP2-Farnesyl-5 were generated by M.W.D. The PH-binding domain of Tubby and AKT fused to GFP (Tubby-EGFP and AKT-EGFP, respectively) were gifts from Tamas Balla. Lifeact-EGFP (Riedl et al., 2008) and CD63-mCherry (derived from CD63-EGFP; Blott et al., 2001) were as previously described.

Cell Culture

OT-I splenocytes were stimulated and grown in T cell medium as previously described (Jenkins et al., 2009). OT-I mice were bred in accordance with approved UK Home Office regulations. Target H-2b EL4 and MC57 stably expressing either Farnesyl-TagBFP2-pMig or TagRFP-MEM-pMig were maintained in DMEM, 10% fetal calf serum (FCS), L-glutamine, 50 U/ml penicillin, and streptomycin (GIBCO).

Immunofluorescence

Glass coverslips (Carl Zeiss) were cleaned in 1M KOH and sonicated for 15 min, coated with poly-L-lysine for 15 min, washed with PBS, coated with 10 μg/ml of either anti-murine CD3ε (clone 145-2C11) or CD25 (clone 3C7) (Becton Dickinson) overnight at 4°C, rinsed in PBS, and blocked with RPMI 10% FCS. 6–8 days after stimulation, OT-I CTLs were added dropwise and allowed to adhere for 3 min. Cells were extracted in 50 mM PIPES, 2.5 mM $MgCl_2$, 2.5 mM EGTA, 0.25% glutaraldehyde, and 0.5% Triton X-100 for 1 min immediately prior to fixation in 4% paraformaldehyde for 15 min. They were then stained with phalloidin-Alexa 488 (Invitrogen), anti-α-tubulin (clone B-5-1-2, Sigma), and Alexa-568-labeled anti-mouse immunoglobulin G (IgG). Images were obtained on a Zeiss Elyra structured illumination microscope and processed with Imaris software (Bitplane).

Live Cell Imaging

5–8 days after stimulation, 5×10^6 to 1×10^7 OT-I CTLs were transfected with 5–7.5 μg DNA with the Mouse T Cell Nucleofector Kit (Lonza) 24 hr prior to imaging. Targets EL4-Farnesyl-5-TagBFP2 and EL4-Mem-TagRFP were pulsed with 1 μM OVA peptide for 1 hr at 37°C and washed twice in serum-free DMEM (GIBCO). Targets were plated on 35-mm glass-bottom culture dishes (MatTek) coated with 1 μg/ml murine ICAM-1/FC (R&D Systems) for 5 min in T cell medium plus 25 mM HEPES. Approximately 2×10^6 CTLs were added dropwise, and imaging started within 5 min. Hoescht 33342 (Invitrogen) was added 20 min earlier for nuclear stain.

For live TIRF microscopy, CTLs (prepared as above) were plated on #1.5 clean, coverglass, 8-well imaging chambers (Lab-Tek) coated with 0.01% poly-L-lysine (Sigma) and then CD3ε in warm T cell media. Imaging was started within 2 min.

Spinning-disc confocal imaging of CTL-target interactions was collected with either an Andor Revolution spinning-disc microscope with a 1.2× camera adaptor or a Nikon Ti3 spinning-disc system with a Photometrics camera at 37°C with a brick-and-block system for temperature and atmosphere regulation. Serial confocal 0.8-μm z stacks were taken at 25- or 20-s intervals, and fluorophores were excited at 405, 488, and 561 nm in each z plane. 4D data sets were rendered and analyzed with Imaris software (Bitplane) and ImageJ.

4D lattice light-sheet measurements were performed as previously described (Chen et al., 2014). Raw data were deconvolved via a 3D iterative Lucy-Richardson algorithm in MATLAB (The Mathworks) via an experimentally measured point spread function. Time-lapse, 3D volumetric data were imported into Amira (FEI) for rendering and visualization.

Analysis

Imaris software (Bitplane) was used for the following analyses. (1) Actin-flow velocity was analyzed with the use of 4D image stacks of data (from the lattice

light-sheet microscope) of CTLs expressing Lifeact-mEmerald as they engaged EL4-red targets. Spatial stability was maintained in the signal by the generation of a 3D isosurface around the 488-nm channel signal, and the centroid was used for correcting for cell drift. Particles were assigned manually to visible actin structures in the 3D rendering of the actin signal over time. Algorithms for tracking native particles were used to connect these particles and generate tracks, and the mean speed of each track was determined. (2) For analyzing average centrosome velocity, the distance between the centrosome and synapse plasma membrane at the time of initial CTL-target contact was divided by the time taken for the centrosome to reach the synapse. (3) Granule-centrosome spatiotemporal relationships were analyzed via tracking native-particle identification for determining xyz coordinate positions for the centrosome and granules at each time point. These data were analyzed by a custom MATLAB program that was written to determine the distance between each granule and the centrosome in 3D for each time point.

SUPPLEMENTAL INFORMATION

Supplemental Information includes three figures and nine movies and can be found with this article online at http://dx.doi.org/10.1016/j.immuni.2015.04.013.

AUTHOR CONTRIBUTIONS

A.T.R., Y.A., and G.M.G. designed the research and wrote the manuscript; A.T.R. performed research and analyzed data for all figures; Y.A. contributed to all microscopy and analyses used in Figures 1, 3, 5, and 6; S.v.E. assisted with data analysis in Figures 2 and 6; J.C.S., N.M.G.D., and C.G.-B. provided additional imaging data for Figures 1, 4, and 7 and responses to review; B.-C.C., W.L., L.G., and E.B. provided acquisition and analysis for lattice light-sheet microscopy in Figure 2; and M.W.D. provided constructs with TagBFP2. Spinning-disc microscopy was carried out in the G.M.G. lab, TIRF and super-resolution microscopy were performed in the J.L.-S. lab, and lattice light-sheet microscopy was performed in the E.B. lab.

ACKNOWLEDGMENTS

Funding was provided by the Wellcome Trust through Principal Research Fellowships (075880 and 103930) to G.M.G. and a Strategic Award (100140) to the Cambridge Institute for Medical Research (CIMR). A.T.R. is an NIH-OxCam scholar supported by funding to J.L.-S. from the Eunice Shriver National Institute of Child Health and Human Development. At the NIH, we thank Senta Kapnick and Pam Schwartzberg for assistance with CTL culture and helpful discussion, P. Sengupta for Farnesyl-EGFP, and Erin Fincher for assistance with the graphic illustration. At the CIMR, we thank Matthew Gratian and Mark Bowen for support with imaging and Alison Schuldt and John James for critical reading of the manuscript. The light-sheet technology used herein is covered by US patent 7,894,136 (issued to E.B. and assigned to Lattice Light LLC of Ashburn, VA), US patent 8,711,211 (issued to E.B. and assigned to the Howard Hughes Medical Institute [HHMI]), and US patent application 13/844,405 (filed by E.B. and assigned to the HHMI).

Received: October 1, 2014
Revised: December 20, 2014
Accepted: March 15, 2015
Published: May 19, 2015

REFERENCES

Babich, A., and Burkhardt, J.K. (2011). Lymphocyte signaling converges on microtubules. Immunity *34*, 825–827.

Babich, A., Li, S., O'Connor, R.S., Milone, M.C., Freedman, B.D., and Burkhardt, J.K. (2012). F-actin polymerization and retrograde flow drive sustained PLCγ1 signaling during T cell activation. J. Cell Biol. *197*, 775–787.

Balagopalan, L., Sherman, E., Barr, V.A., and Samelson, L.E. (2011). Imaging techniques for assaying lymphocyte activation in action. Nat. Rev. Immunol. *11*, 21–33.

Beal, A.M., Anikeeva, N., Varma, R., Cameron, T.O., Vasiliver-Shamis, G., Norris, P.J., Dustin, M.L., and Sykulev, Y. (2009). Kinetics of early T cell receptor signaling regulate the pathway of lytic granule delivery to the secretory domain. Immunity *31*, 632–642.

Beemiller, P., Jacobelli, J., and Krummel, M.F. (2012). Integration of the movement of signaling microclusters with cellular motility in immunological synapses. Nat. Immunol. *13*, 787–795.

Blott, E.J., Bossi, G., Clark, R., Zvelebil, M., and Griffiths, G.M. (2001). Fas ligand is targeted to secretory lysosomes via a proline-rich domain in its cytoplasmic tail. J. Cell Sci. *114*, 2405–2416.

Botelho, R.J., Teruel, M., Dierckman, R., Anderson, R., Wells, A., York, J.D., Meyer, T., and Grinstein, S. (2000). Localized biphasic changes in phosphatidylinositol-4,5-bisphosphate at sites of phagocytosis. J. Cell Biol. *151*, 1353–1368.

Brown, A.C., Oddos, S., Dobbie, I.M., Alakoskela, J.M., Parton, R.M., Eissmann, P., Neil, M.A., Dunsby, C., French, P.M., Davis, I., and Davis, D.M. (2011). Remodelling of cortical actin where lytic granules dock at natural killer cell immune synapses revealed by super-resolution microscopy. PLoS Biol. *9*, e1001152.

Bunnell, S.C., Kapoor, V., Trible, R.P., Zhang, W., and Samelson, L.E. (2001). Dynamic actin polymerization drives T cell receptor-induced spreading: a role for the signal transduction adaptor LAT. Immunity *14*, 315–329.

Burnette, D.T., Manley, S., Sengupta, P., Sougrat, R., Davidson, M.W., Kachar, B., and Lippincott-Schwartz, J. (2011). A role for actin arcs in the leading-edge advance of migrating cells. Nat. Cell Biol. *13*, 371–381.

Campi, G., Varma, R., and Dustin, M.L. (2005). Actin and agonist MHC-peptide complex-dependent T cell receptor microclusters as scaffolds for signaling. J. Exp. Med. *202*, 1031–1036.

Chen, B.C., Legant, W.R., Wang, K., Shao, L., Milkie, D.E., Davidson, M.W., Janetopoulos, C., Wu, X.S., Hammer, J.A., 3rd, Liu, Z., et al. (2014). Lattice light-sheet microscopy: imaging molecules to embryos at high spatiotemporal resolution. Science *346*, 1257998.

Costello, P.S., Gallagher, M., and Cantrell, D.A. (2002). Sustained and dynamic inositol lipid metabolism inside and outside the immunological synapse. Nat. Immunol. *3*, 1082–1089.

Das, V., Nal, B., Dujeancourt, A., Thoulouze, M.I., Galli, T., Roux, P., Dautry-Varsat, A., and Alcover, A. (2004). Activation-induced polarized recycling targets T cell antigen receptors to the immunological synapse; involvement of SNARE complexes. Immunity *20*, 577–588.

Dustin, M.L. (2005). A dynamic view of the immunological synapse. Semin. Immunol. *17*, 400–410.

Freiberg, B.A., Kupfer, H., Maslanik, W., Delli, J., Kappler, J., Zaller, D.M., and Kupfer, A. (2002). Staging and resetting T cell activation in SMACs. Nat. Immunol. *3*, 911–917.

Gao, L., Shao, L., Higgins, C.D., Poulton, J.S., Peifer, M., Davidson, M.W., Wu, X., Goldstein, B., and Betzig, E. (2012). Noninvasive imaging beyond the diffraction limit of 3D dynamics in thickly fluorescent specimens. Cell *151*, 1370–1385.

Giannone, G., Dubin-Thaler, B.J., Rossier, O., Cai, Y., Chaga, O., Jiang, G., Beaver, W., Döbereiner, H.G., Freund, Y., Borisy, G., and Sheetz, M.P. (2007). Lamellipodial actin mechanically links myosin activity with adhesion-site formation. Cell *128*, 561–575.

Gillingham, A.K., and Munro, S. (2000). The PACT domain, a conserved centrosomal targeting motif in the coiled-coil proteins AKAP450 and pericentrin. EMBO Rep. *1*, 524–529.

Gustafsson, M.G. (2000). Surpassing the lateral resolution limit by a factor of two using structured illumination microscopy. J. Microsc. *198*, 82–87.

Hashimoto-Tane, A., Yokosuka, T., Sakata-Sogawa, K., Sakuma, M., Ishihara, C., Tokunaga, M., and Saito, T. (2011). Dynein-driven transport of T cell receptor microclusters regulates immune synapse formation and T cell activation. Immunity *34*, 919–931.

Huse, M., Le Floc'h, A., and Liu, X. (2013). From lipid second messengers to molecular motors: microtubule-organizing center reorientation in T cells. Immunol. Rev. *256*, 95–106.

Ilani, T., Vasiliver-Shamis, G., Vardhana, S., Bretscher, A., and Dustin, M.L. (2009). T cell antigen receptor signaling and immunological synapse stability require myosin IIA. Nat. Immunol. *10*, 531–539.

Janmey, P.A., and Lindberg, U. (2004). Cytoskeletal regulation: rich in lipids. Nat. Rev. Mol. Cell Biol. *5*, 658–666.

Jenkins, M.R., Tsun, A., Stinchcombe, J.C., and Griffiths, G.M. (2009). The strength of T cell receptor signal controls the polarization of cytotoxic machinery to the immunological synapse. Immunity *31*, 621–631.

Jenkins, M.R., Stinchcombe, J.C., Au-Yeung, B.B., Asano, Y., Ritter, A.T., Weiss, A., and Griffiths, G.M. (2014). Distinct structural and catalytic roles for Zap70 in formation of the immunological synapse in CTL. eLife *3*, e01310.

Kaizuka, Y., Douglass, A.D., Varma, R., Dustin, M.L., and Vale, R.D. (2007). Mechanisms for segregating T cell receptor and adhesion molecules during immunological synapse formation in Jurkat T cells. Proc. Natl. Acad. Sci. USA *104*, 20296–20301.

Le Floc'h, A., Tanaka, Y., Bantilan, N.S., Voisinne, G., Altan-Bonnet, G., Fukui, Y., and Huse, M. (2013). Annular PIP3 accumulation controls actin architecture and modulates cytotoxicity at the immunological synapse. J. Exp. Med. *210*, 2721–2737.

Mempel, T.R., Pittet, M.J., Khazaie, K., Weninger, W., Weissleder, R., von Boehmer, H., and von Andrian, U.H. (2006). Regulatory T cells reversibly suppress cytotoxic T cell function independent of effector differentiation. Immunity *25*, 129–141.

Monks, C.R., Freiberg, B.A., Kupfer, H., Sciaky, N., and Kupfer, A. (1998). Three-dimensional segregation of supramolecular activation clusters in T cells. Nature *395*, 82–86.

Poenie, M., Kuhn, J., and Combs, J. (2004). Real-time visualization of the cytoskeleton and effector functions in T cells. Curr. Opin. Immunol. *16*, 428–438.

Porat-Shliom, N., Milberg, O., Masedunskas, A., and Weigert, R. (2013). Multiple roles for the actin cytoskeleton during regulated exocytosis. Cell. Mol. Life Sci. *70*, 2099–2121.

Rak, G.D., Mace, E.M., Banerjee, P.P., Svitkina, T., and Orange, J.S. (2011). Natural killer cell lytic granule secretion occurs through a pervasive actin network at the immune synapse. PLoS Biol. *9*, e1001151.

Raucher, D., Stauffer, T., Chen, W., Shen, K., Guo, S., York, J.D., Sheetz, M.P., and Meyer, T. (2000). Phosphatidylinositol 4,5-bisphosphate functions as a second messenger that regulates cytoskeleton-plasma membrane adhesion. Cell *100*, 221–228.

Riedl, J., Crevenna, A.H., Kessenbrock, K., Yu, J.H., Neukirchen, D., Bista, M., Bradke, F., Jenne, D., Holak, T.A., Werb, Z., et al. (2008). Lifeact: a versatile marker to visualize F-actin. Nat. Methods *5*, 605–607.

Sims, T.N., Soos, T.J., Xenias, H.S., Dubin-Thaler, B., Hofman, J.M., Waite, J.C., Cameron, T.O., Thomas, V.K., Varma, R., Wiggins, C.H., et al. (2007). Opposing effects of PKCtheta and WASp on symmetry breaking and relocation of the immunological synapse. Cell *129*, 773–785.

Soares, H., Henriques, R., Sachse, M., Ventimiglia, L., Alonso, M.A., Zimmer, C., Thoulouze, M.I., and Alcover, A. (2013). Regulated vesicle fusion generates signaling nanoterritories that control T cell activation at the immunological synapse. J. Exp. Med. *210*, 2415–2433.

Stinchcombe, J.C., Barral, D.C., Mules, E.H., Booth, S., Hume, A.N., Machesky, L.M., Seabra, M.C., and Griffiths, G.M. (2001a). Rab27a is required for regulated secretion in cytotoxic T lymphocytes. J. Cell Biol. *152*, 825–834.

Stinchcombe, J.C., Bossi, G., Booth, S., and Griffiths, G.M. (2001b). The immunological synapse of CTL contains a secretory domain and membrane bridges. Immunity *15*, 751–761.

Stinchcombe, J.C., Majorovits, E., Bossi, G., Fuller, S., and Griffiths, G.M. (2006). Centrosome polarization delivers secretory granules to the immunological synapse. Nature *443*, 462–465.

Varma, R., Campi, G., Yokosuka, T., Saito, T., and Dustin, M.L. (2006). T cell receptor-proximal signals are sustained in peripheral microclusters and terminated in the central supramolecular activation cluster. Immunity *25*, 117–127.

Wollman, R., and Meyer, T. (2012). Coordinated oscillations in cortical actin and Ca2+ correlate with cycles of vesicle secretion. Nat. Cell Biol. *14*, 1261–1269.

Yannelli, J.R., Sullivan, J.A., Mandell, G.L., and Engelhard, V.H. (1986). Reorientation and fusion of cytotoxic T lymphocyte granules after interaction with target cells as determined by high resolution cinemicrography. J. Immunol. *136*, 377–382.

Yi, J., Wu, X.S., Crites, T., and Hammer, J.A., 3rd. (2012). Actin retrograde flow and actomyosin II arc contraction drive receptor cluster dynamics at the immunological synapse in Jurkat T cells. Mol. Biol. Cell *23*, 834–852.

Yudushkin, I.A., and Vale, R.D. (2010). Imaging T-cell receptor activation reveals accumulation of tyrosine-phosphorylated CD3ζ in the endosomal compartment. Proc. Natl. Acad. Sci. USA *107*, 22128–22133.

Immunity

Supplemental Information

Actin Depletion Initiates Events Leading to Granule Secretion at the Immunological Synapse

Alex T. Ritter, Yukako Asano, Jane C. Stinchcombe, N.M.G. Dieckmann, Bi-Chang Chen, C. Gawden-Bone, Schuyler van Engelenburg, Wesley Legant, Liang Gao, Michael W. Davidson, Eric Betzig, Jennifer Lippincott-Schwartz, and Gillian M. Griffiths

Figure S1

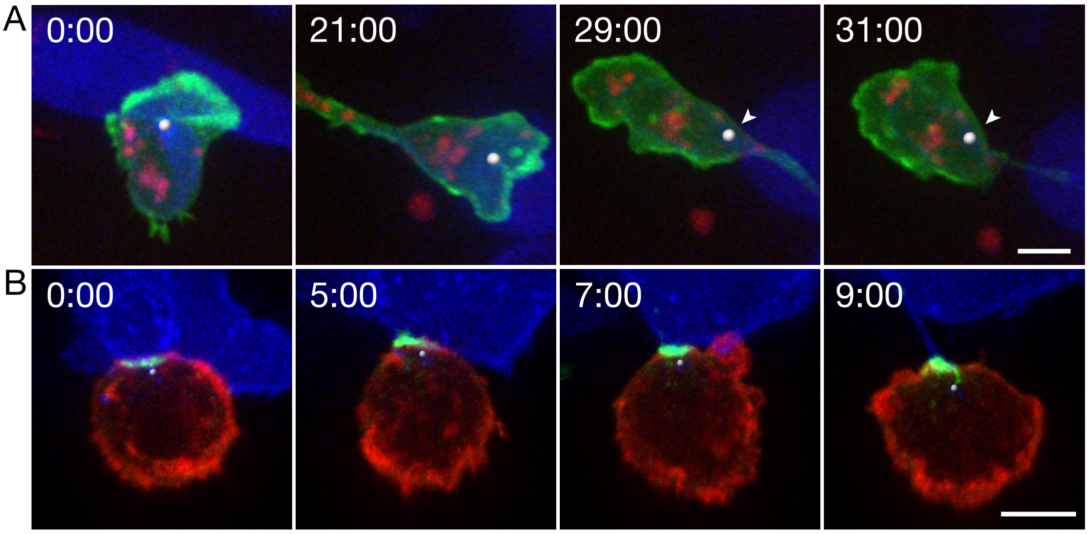

Figure S1: CTL assume the morphology of a migrating cell when retracting from target, related to Figure 6. (A) Timelapse of maximum intensity projection images of a CTL expressing Lifeact-EGFP, CD63-mCherry, and MAPTau-tagBFP2 interacting with a MC57 target cell (blue), (n=13). The location of the centrosome is marked with a white sphere. Arrows highlight the location of the centrosome in the uropod upon retraction. (B) Timelapse of maximum intensity projection images of a CTL expressing CD3ζ-EGFP, Lifeact-mApple and PACT-tagBFP (marked with a white sphere) interacting with an EL4 target cell (blue). Scale bar: 5μm. Time in min**.**

Figure S2

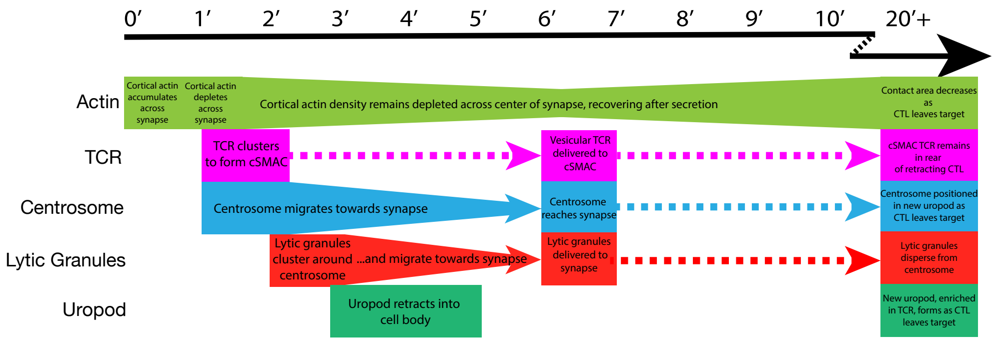

Figure S2, related to Discussion: The order of events leading to secretion at the immunological synapse. Timeline (in min) showing the order of events after a CTL encounters a cognate target with the reorganization of cortical actin, TCR, centrosome, granules and uropod depicted relative to one another as described in the text.

Figure S3

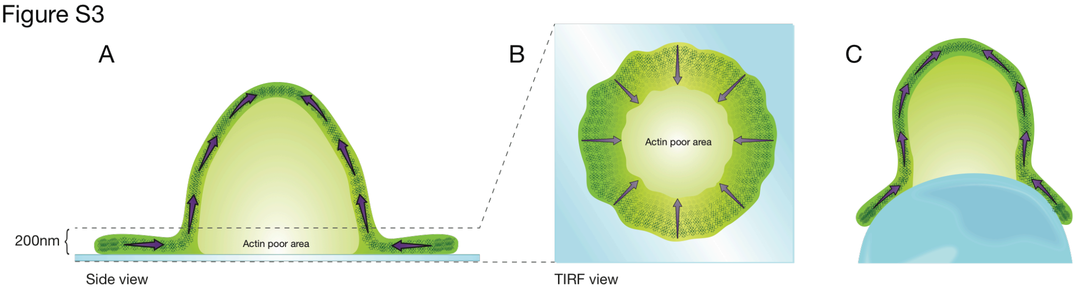

Figure S3: Actin flow in 2D versus 3D summary, related to Discussion. Cartoons to illustrate the flow of cortical actin in CTL cell synapses. (A) Side and (B) TIRF views of a rearward actin flow (purple arrows, away from the synapse) on a planar surface and (C) in a conjugate. By TIRF microscopy, in which only structures within 200nm of the coverslip are illuminated, actin would appear to move centripetally and then disappear. 3D imaging reveals that cortical actin continues to move rearward towards the back of the CTL. See also Movie S9.
